# Community delivery of antiretroviral drugs: A non-inferiority cluster-randomized pragmatic trial in Dar es Salaam, Tanzania

**DOI:** 10.1371/journal.pmed.1002659

**Published:** 2018-09-19

**Authors:** Pascal Geldsetzer, Joel M. Francis, David Sando, Gerda Asmus, Irene A. Lema, Eric Mboggo, Happiness Koda, Sharon Lwezaula, Ramya Ambikapathi, Wafaie Fawzi, Nzovu Ulenga, Till Bärnighausen

**Affiliations:** 1 Department of Global Health and Population, Harvard T.H. Chan School of Public Health, Harvard University, Boston, Massachusetts, United States of America; 2 Management and Development for Health, Dar es Salaam, Tanzania; 3 Faculty of Economics and Social Sciences, Heidelberg University, Heidelberg, Germany; 4 Tanzanian National AIDS Control Programme, Ministry of Health, Community Development, Gender, Elderly and Children, Dar es Salaam, Tanzania; 5 Department of Nutrition, Harvard T.H. Chan School of Public Health, Harvard University, Boston, Massachusetts, United States of America; 6 Department of Epidemiology, Harvard T.H. Chan School of Public Health, Harvard University, Boston, Massachusetts, United States of America; 7 Heidelberg Institute of Global Health, Heidelberg University, Heidelberg, Germany; 8 Africa Health Research Institute, KwaZulu-Natal, South Africa; University of Southampton, UNITED KINGDOM

## Abstract

**Background:**

With the increase in people living with HIV in sub-Saharan Africa and expanding eligibility criteria for antiretroviral therapy (ART), there is intense interest in the use of novel delivery models that allow understaffed health systems to successfully deal with an increasing demand for antiretroviral drugs (ARVs). This pragmatic randomized controlled trial in Dar es Salaam, Tanzania, evaluated a novel model of ARV community delivery: lay health workers (home-based carers [HBCs]) deliver ARVs to the homes of patients who are clinically stable on ART, while nurses and physicians deliver standard facility-based care for patients who are clinically unstable. Specifically, the trial aimed to assess whether the ARV community delivery model performed at least equally well in averting virological failure as the standard of care (facility-based care for all ART patients).

**Methods and findings:**

The study took place from March 1, 2016, to October 27, 2017. All (48) healthcare facilities in Dar es Salaam that provided ART and had an affiliated team of public-sector HBCs were randomized 1:1 to either (i) ARV community delivery (intervention) or (ii) the standard of care (control). Our prespecified primary endpoint was the proportion of adult non-pregnant ART patients with virological failure at the end of the study period. The prespecified margin of non-inferiority was a risk ratio (RR) of 1.45. The mean follow-up period was 326 days. We obtained intent-to-treat (ITT) RRs using a log-binomial model adjusting standard errors for clustering at the level of the healthcare facility. A total of 2,172 patients were enrolled at intervention (1,163 patients) and control (1,009 patients) facilities. Of the 1,163 patients in the intervention arm, 516 (44.4%) were both clinically stable on ART and opted to receive ARVs in their homes or at another meeting point of their choosing in the community. At the end of the study period, 10.9% (95/872) of patients in the control arm and 9.7% (91/943) in the intervention arm were failing virologically. The ITT RR for virological failure demonstrated non-inferiority of the ARV community delivery model (RR 0.89 [1-sided 95% CI 0.00–1.18]). We observed no significant difference between study arms in self-reported patient healthcare expenditures over the last 6 months before study exit. Of those who received ARVs in the community, 97.2% (95% CI 94.7%–98.7%) reported being either “satisfied” or “very satisfied” with the program. Other than loss to follow-up (18.9% in the intervention and 13.6% in the control arm), the main limitation of this trial was that substantial decongestion of healthcare facilities was not achieved, thus making the logic for our preregistered ITT approach (which includes those ineligible to receive ARVs at home in the intervention sample) less compelling.

**Conclusions:**

In this study, an ARV community delivery model performed at least as well as the standard of care regarding the critical health indicator of virological failure. The intervention did not significantly reduce patient healthcare expenditures, but satisfaction with the program was high and it is likely to save patients time. Policy-makers should consider piloting, evaluating, and scaling more ambitious ARV community delivery programs that can reach higher proportions of ART patients.

**Trial registration:**

ClinicalTrials.gov NCT02711293.

## Abstract 2

In a cluster-randomized pragmatic trial from Tanzania, Pascal Geldsetzer and colleagues investigate different delivery models of antiretroviral drugs to HIV patients at home compared to standard facility-based care.

## Author summary

**Why was this study done?:**

- The number of individuals in sub-Saharan Africa needing antiretroviral therapy (ART) for HIV—and chronic disease care more broadly—is expected to increase over the coming decades, further straining already under-resourced health systems in the region.
- Community delivery of antiretroviral drugs (ARVs) has the potential to decrease ART patient volume at healthcare facilities and reduce patient and government healthcare expenditures, while maintaining the positive effects of ART on health outcomes.

**What did the researchers do and find?:**

- We randomized 48 ART facilities in Dar es Salaam, Tanzania, 1:1 to (i) ARV community delivery (lay health workers deliver ARVs to the homes of patients who are clinically stable on ART while nurses and physicians deliver standard facility-based care for patients who are clinically unstable on ART) or (ii) control (standard facility-based care for all patients).
- We measured whether the risk of virological failure (i.e., poor control of one’s HIV infection) in the ARV community delivery arm was lower than, or equal to that in the control arm using a prespecified threshold above which we considered the intervention to be inferior to the standard of care.
- As implemented in this study, in which roughly 40% of patients enrolled in the intervention arm received ARVs at home at least once and 60% remained in standard facility-based care, the new ARV community delivery model performed at least as well as the standard of care regarding the critical health indicator of virological failure.
- While we did not observe any differences in patient healthcare expenditures between the 2 arms of the study, patients’ satisfaction with receiving ARVs at home was high.

**What do these findings mean?:**

- While ARV community delivery appears to have been safe with respect to controlling patients’ HIV infection, some of the expected benefits of the program—decongestion of healthcare facilities and reduction in patients’ healthcare expenditures—were not realized.
- Nonetheless, the program was popular with patients, presumably because it makes ART care more convenient and saves patients time.
- Policy-makers should consider piloting, scaling, and evaluating more ambitious ARV community delivery programs that reach higher proportions of ART patients than reached in this trial.

## Introduction

Chronic diseases are rapidly replacing acute infectious diseases as the leading cause of the disease burden in sub-Saharan Africa (SSA) [1]. While many of these chronic conditions are non-communicable, HIV has also become a chronic illness as effective therapy allows HIV-positive individuals to survive into old age [2,3]. Indeed, the HIV epidemic continues to take a considerable toll in SSA, where approximately 25.5 million people were living with HIV in 2016 [4]. This shift to chronic conditions poses new challenges to generally weak health systems in SSA.

HIV, in particular, is expected to place an increasing stress on health systems and patients in SSA in the coming years. The World Health Organization (WHO) eliminated all CD4 cell count treatment thresholds in its 2016 HIV treatment guidelines, recommending antiretroviral therapy (ART) for all people living with HIV [5]. As countries are gradually starting to implement ART for all, this will likely lead to a substantial rise in the number of people on ART in SSA in the coming years [6], particularly if guideline changes are coupled with an increased identification of individuals living with HIV who are currently unaware of their HIV status. Under former HIV care guidelines, HIV patients on ART generally attended facility-based care at least twice as frequently as HIV patients who were not yet on ART [7]. Given the high prevalence of HIV across SSA, the implementation of the new WHO treatment guidelines is thus expected to place further strain on nurses and physicians, as well as increase patient healthcare expenditures [8].

Community delivery of antiretroviral drugs (ARVs) through lay health workers is a promising ART delivery model, because it should increase the capacity of the health system to effectively provide care to rising ART patient numbers [5]. ARV community delivery may not only ease the workload of nurses and physicians (e.g., reducing the frequency with which ART patients have to attend healthcare facilities), but may also improve ART adherence and retention among patients (e.g., by increasing the convenience of remaining in care) [9]. In addition, it is plausible that ARV community delivery can reduce patient healthcare expenditures, because patients receive treatment in or close to their homes and thus save the time and financial costs of travel to healthcare facilities [10]. On the other hand, ARV community delivery may have adverse consequences for ART patients’ health by decreasing the frequency with which patients are seen by more highly trained healthcare workers.

This non-inferiority cluster-randomized pragmatic trial aimed to establish the effectiveness of ARV community delivery when implemented in the routine healthcare system of Dar es Salaam, the largest city in East Africa [11]. Specifically, this study aimed to determine whether an ARV community delivery model (lay health workers deliver ARVs to the homes of patients who are clinically stable on ART and nurses and physicians deliver standard facility-based care for patients who are clinically unstable on ART) leads to a lower or equal (“non-inferior”) risk of virological failure compared to the standard of care (standard facility-based care for all ART patients). A secondary aim of this study was to determine the impact of the ARV community delivery model on patient healthcare expenditures. This study was registered in the clinical trials registry of the United States National Library of Medicine at the National Institutes of Health, ClinicalTrials.gov (NCT02711293).

## Methods

A detailed protocol of this study is available in the public domain [12].

### Study setting

This study took place in all 3 municipalities (Temeke, Kinondoni, and Ilala) of Dar es Salaam. Dar es Salaam is the most urbanized region of Tanzania, with an estimated 5.4 million inhabitants in 2016. The population of the city is expected to grow to 10.8 million by 2030 [11]. In 2012, when the latest HIV/AIDS and Malaria Indicator Survey was carried out in Tanzania, Dar es Salaam’s HIV prevalence was estimated to be 6.9% among adults aged 15–49 years, while the national prevalence was 5.1% [13]. The most recent HIV treatment guidelines in Tanzania were published in May 2015 (i.e., prior to trial start) and recommend initiation of ART for adults if the patient has a CD4 cell count < 500 cells/μl or is in WHO stage 3 or 4 [14].

### Tanzania’s home-based carer program

Home-based carers (HBCs) are a lay health worker cadre in Tanzania’s public-sector health system. There are approximately 35,000 HBCs in Tanzania, and the program has existed since 1996. HBCs work in the neighborhoods in which they live; 1 to 3 HBCs serve one neighborhood. The HBC program exists in most, but not all, neighborhoods of Dar es Salaam. HBCs’ main responsibility consists of conducting regular (at least every 3 months) visits to HIV patients’ households in their assigned neighborhood. Their tasks have varied over the years but generally consist of household visits to provide counseling on ARV adherence, family planning, and nutrition; to promote the uptake of preventive healthcare services; and to refer ill clients to a healthcare facility. HBCs are affiliated with 1 healthcare facility in the vicinity of their neighborhood. A facility-based nurse (“community outreach nurse”) is responsible for supervising the healthcare facility’s team of HBCs. Dar es Salaam’s municipalities pay HBCs a monthly stipend of 50,000 Tanzanian Shillings (TZS) (72 purchasing power parity-adjusted dollars [PPP$]). As part of this trial, HBCs in the intervention arm received a further TZS 75,000 (PPP$ 109) flat payment per month to compensate them for the additional transport costs and workload. Because HBCs had a varying number of ART clients for home delivery of ARVs, this payment was changed to a payment of 10,000 Tanzanian Shillings (PPP$ 14) per community ARV delivery visit in January 2017.

### Description of the intervention

In clusters randomized to ARV community delivery, patients who were clinically stable on ART could choose to receive ARVs and ART counseling in or close to their homes instead of having to return to the healthcare facility for a clinical checkup and to pick up their next ARV supplies. An HBC visited patients at home or at another meeting point in the community at which the patient wanted to receive his or her ARVs (such as somewhere close to the patient’s workplace). The HBC provided counseling, delivered a supply of ARVs, and performed an ARV pill count. HBC visits were conducted with the same frequency as the patient’s schedule for attending facility-based ART delivery prior to study enrollment, which was either a monthly or 2-monthly HIV care visit. Patients in the intervention arm did not have to return to a facility for HIV care until the study exit assessment. HBCs in the intervention arm received 3 days of training in the community delivery of ARVs and in counseling skills for this intervention prior to the start of the trial. Counseling focused on ART adherence, family planning, prevention of onward HIV transmission, and basic nutrition.

### Eligibility criteria

The eligibility criteria for enrollment into this trial were (i) age ≥ 18 years, (ii) having attended one of the participating healthcare facilities for ART delivery during the enrollment period, and (iii) residing in a neighborhood in the facility’s catchment area (ascertained through self-report). Exclusion criteria were pregnancy at the time of enrollment (ascertained through self-report) and inability to provide written informed consent. Pregnancy was an exclusion criterion because at many of the study’s healthcare facilities, pregnant women with HIV were seen in different sections of the healthcare facility than non-pregnant ART patients. Thus, enrollment of pregnant women would have required additional human resources, which were not available.

The random assignment to ARV community delivery versus standard facility-based care was at the facility level. After enrollment into the trial, patients who attended trial clinics in the control arm continued to receive the standard of care. In the ARV community delivery intervention arm, enrolled patients had to be clinically stable on ART to be eligible to receive ARVs in their homes or at other community meeting points. In consultation with Tanzania’s National AIDS Control Programme, the following criteria were established to define clinical stability on ART: (i) having taken ARVs for at least 6 months prior to study enrollment, (ii) having had a CD4 cell count >350 cells/μl or a suppressed viral load (VL) at 6 or more months after ART initiation (if both a CD4 cell count and VL measurement were taken 6 or more months after ART initiation, then at least 1 CD4 cell count had to be >350 cells/μl and 1 VL measurement had to show virological suppression [<1,000 copies/ml]), and (iii) the most recent VL was taken less than 12 months prior to study enrollment and showed virological suppression. For patients for whom a VL measurement was unavailable at the time of enrollment but for whom a CD4 cell count taken in the 12 months prior to enrollment was available, a CD4 cell count >350 cells/μl was used to replace criterion (iii). For patients for whom neither a VL nor a CD4 cell count taken in the 12 months prior to enrollment was available, a venous blood sample was taken for a VL measurement at the time of enrollment, and the result was used for the eligibility assessment. Type of ARV regimen and co-infection status were not exclusion criteria for receiving ARVs at home. While patients in the control arm underwent the same assessment for clinical stability as those in the intervention arm, all patients in the control arm received standard facility-based HIV care regardless of clinical stability on ART.

### Enrollment

The enrollment process described below was the same in both control and intervention facilities. In each healthcare facility in this trial, 1 to 2 study team members were present full-time for the duration of the periods of enrollment and study exit assessment. During the follow-up period, most data collectors split their time between 3 facilities (1 in each of the 3 municipalities). The ART nurses asked all ART patients attending a participating healthcare facility during the enrollment period whether they resided within the neighborhoods that are part of the facility’s catchment area. Those who reported living in the catchment area were sent to the data collector, who was then responsible for introducing the study to the potential participants and obtaining written informed consent. Provided written informed consent was given, the data collector then administered a tablet-based baseline questionnaire (see the “data collection” section for more details). Next, the data collector noted down a description of the location of the patient’s residence (a “map cue”) and recorded the patient’s mobile phone number (and, with the permission of the patient, the mobile phone number of at least 1 household member). Lastly, for those patients who did not have a VL measurement taken in the 12 months preceding study enrollment, the data collector referred the patient back to the nurse for a blood sample that was then sent to the laboratory for a VL assessment. The only difference in the enrollment process between control and intervention facilities was that in intervention facilities the community outreach nurse was responsible for ensuring that the HBC received the map cue and phone numbers so that the patient could be visited at home.

### Randomization

This was a 2-arm non-inferiority cluster-randomized pragmatic trial. The unit of randomization was a healthcare facility with its catchment area (henceforth referred to as a “cluster”). All healthcare facilities that were located in Dar es Salaam Region, provided ART, and had an affiliated team of HBCs were eligible to be included in the trial. The study took place at all 50 healthcare facilities that fulfilled these eligibility criteria except 2 facilities (Amana Regional Referral Hospital and Mwananyamala Regional Referral Hospital), which were excluded because of an ongoing clinical trial at these sites. S1 Table describes the characteristics of each cluster.

Within each of the 3 municipalities in Dar es Salaam, we first matched clusters into pairs based on the number of patients currently on ART at the healthcare facility. For instance, the healthcare facility with the highest number of ART patients in the municipality of Kinondoni was paired with the facility with the second highest number of ART patients in Kinondoni, and so on. We expected this matched-pair design to increase the precision of our effect estimates because the complexity of implementing the intervention (and, thus, the expected probability of implementation failure) would tend to increase with a higher volume of eligible patients. Specifically, because each healthcare facility had only 1 community outreach nurse (except Kigamboni Health Centre and Mbezi Dispensary, which had 2 community outreach nurses), a higher number of ART patients at the healthcare facility resulted in a higher number of patients for whom the community outreach nurse had to supervise the delivery of ARVs into the community. A second advantage of matching on ART patient volume, and thus expected participant volume, was that it increased the probability of an approximately equal number of participants in each study arm, which in turn maximizes statistical power. The randomization was done (by PG) prior to study start using computer-generated random numbers. Allocation concealment in this trial was achieved because entire healthcare facilities (and thus automatically all eligible patients at these healthcare facilities) were randomized to each study arm, and the team member who randomized healthcare facilities did not enroll patients. It was not feasible to blind study participants, project managers, or data collectors to the intervention assignment. Neither was it possible to blind the data analysts (PG, GA, and TB) to the intervention assignment because they were also involved in the project management.

### Study duration

Enrollment into the trial took place at the 18 healthcare facilities in Temeke municipality from March 1, 2016, to July 29, 2016, at the 16 healthcare facilities in Kinondoni municipality from August 1, 2016, to October 31, 2016, and in the 14 healthcare facilities in Ilala municipality from November 1, 2016, to January 31, 2017. Study exit assessments started in Temeke in March 2017, in Kinondoni in May 2017, and in Ilala in June 2017. The study activities during the trial period are described in S2 Table. There was a delay of 11 calendar days between study start and registration of the study on ClinicalTrials.gov because the first 9 days of the trial were used to verify whether the enrollment process was feasible. This informal pilot period did not lead to any changes in any of the planned study processes and was thus considered to be part of the main trial period. No outcome data were collected before registration of the trial.

### Endpoints

The prespecified primary endpoint for this trial was the proportion of patients with virological failure at the end of the study period. Virological failure was defined as a VL ≥ 1,000 copies/ml. The prespecified secondary endpoint was patient healthcare expenditures in the 6 months preceding study exit.

### Margin of non-inferiority

This study was designed as a non-inferiority trial. The non-inferiority design only applies to the primary endpoint (the proportion of patients with virological failure) [12]. The rationale for choosing a non-inferiority design for this study was that if the intervention results in equivalent (or better) control of one’s HIV infection among ART patients (as assessed through the VL measurement), then the intervention will be preferable to the standard of care because it has several important advantages beyond clinical effectiveness. First, HBCs earn lower salaries and are quicker to train than nurses and physicians. Shifting important components of ART from nurses and physicians to HBCs will thus likely reduce the per-patient costs of ART delivery and increase the capacity to quickly scale up treatment [15]. Second, shifting care from facilities to homes will decongest the facilities and allow nurses and physicians to concentrate on more complex and clinically unstable ART patients who require more intensive clinical workup and care. Finally, ARV community delivery should reduce the financial and time burdens on patients of having to attend a healthcare facility at frequent intervals [8,10].

Based on consultations with Tanzania’s National AIDS Control Programme, and in line with the margin of equivalence used by Jaffar et al. in their randomized trial of ARV home delivery in rural Uganda [10], we chose a margin of non-inferiority for the risk ratio (RR) of virological failure (comparing the intervention to the control arm) of 1.45. That is, if the RR of virological failure in the intervention group compared to the control group is statistically significantly lower than 1.45, then ARV community delivery will be considered to be non-inferior to standard facility-based care. We registered this prespecified margin of non-inferiority as part of our trial protocol in ClinicalTrials.gov. On the absolute scale, this non-inferiority margin corresponds to a higher absolute probability of virological failure in the intervention group of 9 percentage points, assuming (as done by Jaffar et al. [10]) that 20% of patients in the control arm of the study will be failing virologically at the end of the follow-up period.

### Data collection

This trial was implemented by Management and Development for Health (MDH). MDH is a local Tanzanian-led non-governmental organization based in Dar es Salaam, which works in close collaboration with the Tanzanian Ministry of Health, Community Development, Gender, Elderly and Children.

#### Biomarkers

We measured HIV VL at baseline and at the end of the study period. If the patient had a VL measurement that was taken in the 12 months preceding study enrollment (as described under the eligibility criteria), the value of this measurement was used as baseline VL. VL measurements were done at the public-sector laboratory in Temeke using Cobas AmpliPrep TaqMan 96 and Cobas 4800 analyzers.

#### Questionnaires

Two questionnaires—one during the enrollment visit (baseline questionnaire) and one during the study exit assessment visit (study exit questionnaire)—were administered in Swahili (with responses entered into a tablet) by a team of 20 data collectors. Most questions were identical in the 2 questionnaires. Topics covered in the questionnaire included basic socio-demographic information, health service utilization, out-of-pocket healthcare expenditures, and satisfaction with HBC services. Specifically, healthcare expenditures were assessed in 2 ways. First, participants were asked about the costs incurred to attend ART on the day of the study enrollment and study exit visit, separately for each of the following cost components: consultation fees, medical tests, medicines, transport, payment for someone to look after the patient's children while the patient was away, food, phone calls and SMS messages, and income lost due to the time spent to attend the healthcare facility. Second, in both the baseline and study exit questionnaire, the same cost components (except income lost due to the time spent to attend the healthcare facility) were asked about for each primary healthcare visit that the patient had made during the preceding 6 months to each of the following types of providers: public-sector primary care clinic, private-sector doctor, chemist/pharmacy, traditional healer, diviner, and faith healer.

### Ethics and policy

The study was approved by the research ethics committee of the National Institute for Medical Research (NIMR) in Tanzania on July 16, 2015 (NIMR/HQ/R.8a/Vol. IX/1989), and received an exemption by the institutional review board of the Harvard T.H. Chan School of Public Health in June 2015. During the study period, information on trial progress and analyses of the baseline data were shared at least once every 6 months with the National AIDS Control Programme within the Tanzanian Ministry of Health, Community Development, Gender, Elderly and Children. The Coordinator for Care, Treatment and Support in the Tanzanian National AIDS Control Programme (SL) served as a member of the core team that oversaw and managed this trial.

### Sample size

We calculated the sample size needed for this non-inferiority design under individual randomization (using the “ssi” package in Stata [16]), and then multiplied the sample size under individual randomization by the design effect to arrive at the sample size needed for the non-inferiority design under cluster randomization. The design effect was computed using the “clustersampsi” function in Stata [17], which implements a standard method for calculating power in cluster-randomized trials (but does not allow direct sample size calculation for non-inferiority designs). We assumed an intra-cluster correlation coefficient (ICC) of 0.03. This assumption was based on a study in Dar es Salaam [18], which found the healthcare facility ICC value for the 6-month cumulative incidence of non-adherence to ARVs (defined as a 50% drop in CD4 cell count from its peak value and return to pre-ART CD4 cell count or lower after 168 days on ART, or a VL greater than 10,000 copies/ml after 168 days on ART) to be 0.016. We took the upper bound of the 95% CI of this estimate (which was 0.03) as a conservative estimate of the ICC for our primary endpoint. To our knowledge, this was the best approximation of the expected ICC for our primary endpoint available in the extant literature.

Additionally, we made the following assumptions: 20% of patients in the standard of care arm will be failing virologically at study exit, the probability of a type I statistical error is 0.05, and the correlation coefficient between baseline and study exit VL measurement is 0.5. Under these assumptions and our prespecified margin of non-inferiority, this trial needed 398 patients per study arm to have 80% power to establish non-inferiority. We did not stop enrolling patients once the minimum sample size was reached because an important aim of this study was to investigate to what degree the ARV community delivery model could decongest ART facilities. Our actual sample size was thus substantially larger.

### Statistical analysis

The primary analysis in this study was an intent-to-treat (ITT) analysis. All patients at a healthcare facility were in the ITT sample, regardless of whether they were clinically stable on ART and chose to receive ARVs in (or close to) their homes or not. In secondary analyses, we also examined the treatment effects among only those patients who had a suppressed VL (or, if no VL value was available, a CD4 cell count >350 cells/μl) at baseline (henceforth referred to as “suppressed VL at baseline” for simplicity). The effect of the intervention on our prespecified primary endpoint was examined using a log-binomial model, which generates a RR. In this cluster-randomized trial, the odds ratio (OR) and RR are equally valid relative measures of association, but the OR is sometimes misinterpreted as a RR while the reverse is rarely the case [19]. We thus preferred to express our results using a RR. Whether or not the RR was below the non-inferiority margin was assessed using the upper bound of a 1-sided 95% CI. If the upper bound of this CI for the RR comparing intervention to control was below 1.45, the intervention would be deemed non-inferior to the control. If the upper bound of this CI was greater than or equal to 1.45, then the null hypothesis that the intervention was inferior to the control could not be rejected (at the alpha equal to 0.05 level), and the results of the trial would thus be inconclusive. The CI was obtained from the log-binomial model adjusting standard errors for clustering at the level of the healthcare facility. In the primary analysis, we regressed virological failure at study exit on a binary variable indicating intervention versus control assignment. In secondary analyses, we first added VL at baseline as a covariate to our regression and then additionally controlled for, follow-up time, time between the baseline VL and the study exit VL, age, and sex. Our models did not include an indicator variable for each pair of healthcare facilities to adjust for the matched-pair design, because this is not necessary to obtain valid point estimates [20]. In expectation, including an indicator variable for each pair would lead to a lower (or equal) variance than when ignoring the matched-pair design [20]. However, in our case, this possible increase in statistical efficiency was offset by the fact that some pairs are dropped from the regression that includes all covariates if an indicator variable for each pair is used, because some covariate combinations have 0 observations. In addition to the ITT effect, we estimated the complier average causal effect, i.e., the effect of the ARV community delivery model on those patients who actually received ARVs at home. For this purpose, we used instrumental-variable regression with the randomly assigned intervention status of the healthcare facilities participating in this trial as the instrumental variable for patients' potentially endogenous receipt of ARVs in their homes.

The secondary endpoint (patient healthcare expenditures during the last 6 months) was analyzed using ordinary least squares regression (for inference based on the mean expenditure) and median regression (for inference based on the median expenditure). In these regressions, we determined statistical significance using randomization inference (as implemented in the “ritest” Stata package [21]). We chose to use randomization inference to assess statistical significance because it does not rely on asymptotic properties, which may not apply in cluster-randomized trials with a relatively small number of clusters, especially when there is substantial heterogeneity in cluster size [22]. By specifying the randomization scheme of the study, the randomization inference routine adjusted for clustering at the level of the healthcare facility as well as the matched-pair design.

## Results

### Sample characteristics

**Fig 1:**
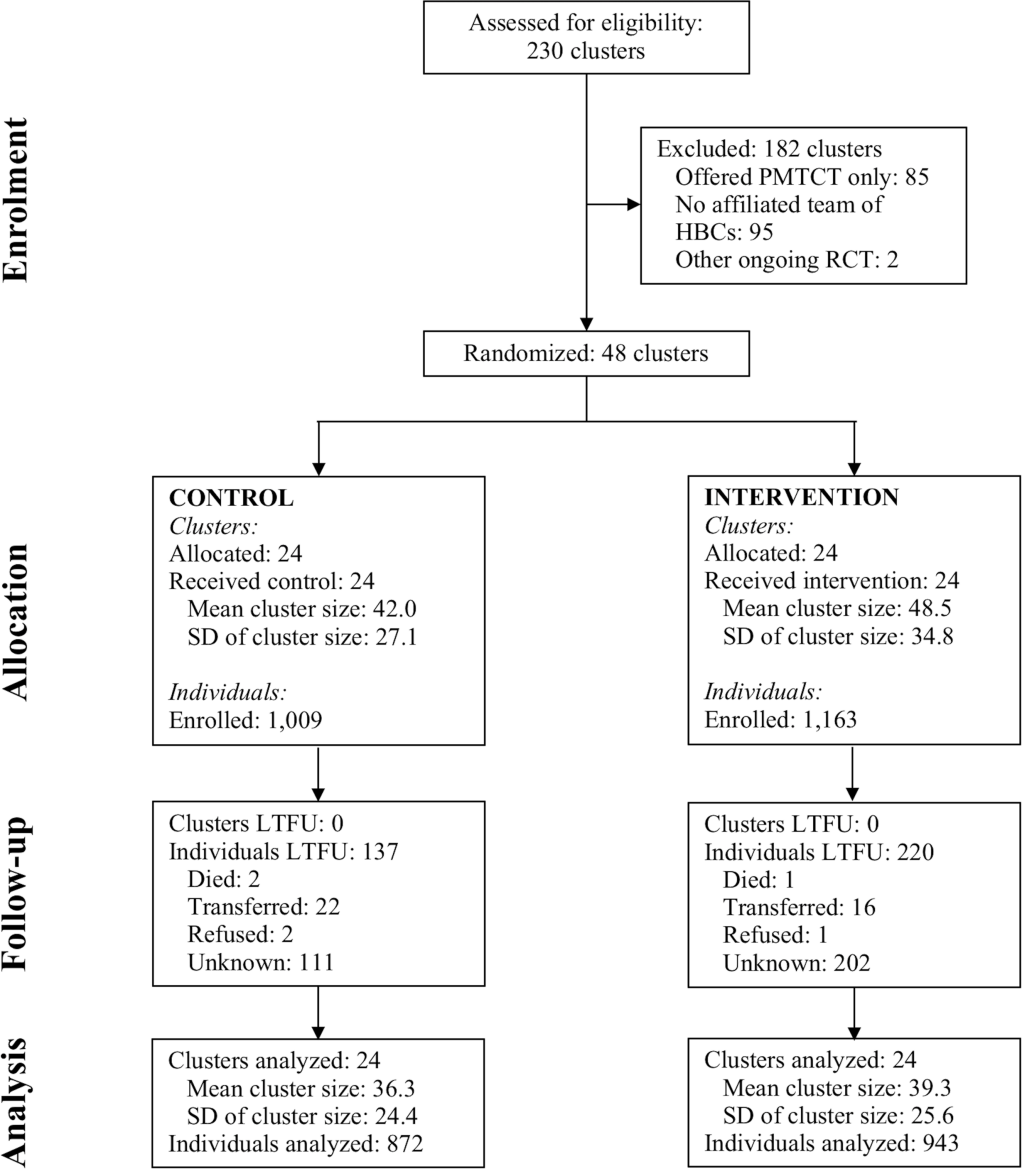
Flowchart showing progression of clusters and patients through the trial. Mean cluster size was rounded to 1 decimal place, which is responsible for the minor discrepancy between the number of individuals enrolled/analyzed and the multiplication of the number of clusters by the mean cluster size. HBC, home-based carer; LTFU, lost to follow-up; PMTCT, prevention of mother-to-child transmission of HIV; RCT, randomized controlled trial; SD, standard deviation.

Fig 1 shows the progression of healthcare facilities (clusters) and patients through the trial. Forty-eight healthcare facilities and 2,172 patients were enrolled into the trial. Twenty-four healthcare facilities with a total of 1,009 patients were enrolled in the control arm (standard facility-based care), and 24 healthcare facilities with a total of 1,163 patients in the intervention arm. The intervention, ARV community delivery, included HBCs delivering ARVs in or close to the homes of patients who were clinically stable on ART. Patients in the intervention arm who were either clinically unstable on ART or who opted not to have their ARVs delivered to their homes continued to receive standard facility-based ART. In all, 516 (44.4%) of the patients enrolled in the intervention arm received ARVs in or close to their homes. For 63 (12.2%) of the patients receiving ARVs in or close to their homes, no VL was available after enrollment (these patients were therefore considered lost to follow-up [LTFU]). For a further 69 (13.4%) of these patients, the only available VL after enrollment was taken prior to receiving the first ARV home visit. These patients were kept in the sample for the primary analysis because they may have indirectly benefited from other patients in their healthcare facility receiving ARVs in or close to their homes. However, we also show results when restricting the sample to those patients receiving ARVs in or close to their homes for at least 90 and 180 days. Among the 359 patients for whom we had a study exit VL taken after receiving the first ARV home visit, the mean duration of receiving ARVs in or close to the home was 226 days, with a standard deviation (SD) of 123 days. The median duration of receiving ARVs in or close to the home was 213 days, with an interquartile range (IQR) of 138 to 300 days.

Thirty-five patients (6 of whom were LTFU) received ARVs in or close to their homes but did not continue until the end of the trial period—4 patients transferred to a healthcare facility outside of Dar es Salaam; 8 informed the study team that they wanted to return to standard facility-based care; 3 were returned to standard facility-based care because they were enrolled based on a baseline CD4 cell count >350 cells/μl but the VL taken at enrollment came back from the laboratory as being non-suppressed; 1 died; 1 was imprisoned; 3 became pregnant and entered into the PMTCT program (without ARV community delivery); and the remainder could not be found again by the HBC. Among the 8 intervention recipients receiving ARVs in or close to their homes who wanted to return to facility-based care, 3 informed the study team that the HBC (the same HBC for all 3 patients) had not delivered their ARVs on time, and the other 5 wanted to return because they were attending the healthcare facility regularly for their children’s healthcare, which made it convenient to pick up ARVs while at the healthcare facility. The 1 death that occurred was a road traffic accident, which was unrelated to the intervention or the trial. Apart from the death, no adverse events were reported to, or detected by, the study team among those patients who received their ARVs in or close to their homes. Similarly, no adverse events among other participants enrolled in this trial (i.e., those in the control arm and those in the intervention arm who did not receive ARVs at home) were reported to the study team. In all, 13.6% (137/1,009) were LTFU in the control arm, and 18.9% (220/1,163) in the intervention arm, yielding a sample size for analysis of 872 patients in the control arm and 943 in the intervention arm.

The sample characteristics for clusters (a healthcare facility with its catchment area) are shown in S1 Table. Table 1 displays the sample characteristics of individuals at the time of the baseline assessment. Patients in the intervention arm were somewhat more likely to be male (22.2% versus 15.4%), to be married (44.3% versus 35.8%), and to self-report having been on ART for a longer time at baseline (mean of 1,407 versus 1,059 days). The percentage failing virologically at baseline was similar between the 2 study arms. The mean follow-up time was 326 days (SD 125) in the control arm and 327 days (SD 120) in the intervention arm. Median follow-up time was also similar between the study arms: 318 days in the control and 322 days in the intervention arm. S3 Table shows that the baseline characteristics of those who were LTFU were similar to those who were included in the analysis except that they were (i) less likely to have received ARVs at home (intervention arm: 28.6% versus 48.0%) and (ii) more likely to have been failing virologically (control arm: 28.2% versus 17.4%; intervention arm: 19.8% versus 15.4%).

**Table 1:** Sample characteristics at baseline among patients not lost to follow-up.

Characteristic	Control	Intervention
***N***	872	943
**Male, *n* (%)**	129 (15.4)	203 (22.2)
*Missing*, *n* (%)	33 (3.8)	30 (3.2)
**Age in years, mean (SD)**	38.7 (8.6)	40.5 (9.4)
*Missing*, *n* (%)	40 (4.6)	35 (3.7)
**Age group, *n* (%)**		
18–25 years	41 (4.9)	32 (3.5)
26–35 years	260 (31.2)	259 (28.5)
36–45 years	371 (44.6)	384 (42.3)
46–55 years	129 (15.5)	171 (18.8)
56–65 years	25 (3.0)	53 (5.8)
>65 years	6 (0.7)	9 (1.0)
**Education, *n* (%)**		
Less than completed primary school	26 (4.3)	66 (9.8)
Primary school completed	473 (77.7)	512 (76.1)
Secondary school completed	110 (18.1)	95 (14.1)
*Missing*, *n* (%)	263 (30.2)	270 (28.6)
**Married, *n* (%)**	237 (35.8)	334 (44.3)
*Missing*, *n* (%)	210 (24.1)	189 (20.0)
**Time on ART in days, mean (SD)**	1,059 (952)	1,407 (1171)
*Missing*, *n* (%)	277 (31.8)	304 (32.2)
**Time on ART, *n* (%)**		
<90 days	57 (9.6)	48 (7.5)
90–179 days	34 (5.7)	19 (3.0)
180–364 days	73 (12.3)	58 (9.1)
1 to <3 years	210 (35.3)	202 (31.6)
3 to <5 years	109 (18.3)	121 (18.9)
≥5 years	112 (18.8)	191 (29.9)
**Disclosed HIV status to at least 1 person, *n* (%)**	542 (88.4)	625 (92.0)
*Missing*, *n* (%)	259 (29.7)	264 (28.0)
**VL ≥1,000 copies/ml or CD4 <350 cells/**μ**l, *n* (%)**	132 (17.4)	122 (15.4)
*Missing*, *n* (%)	114 (13.1)	150 (15.9)

ART, antiretroviral therapy; SD, standard deviation; VL, viral load.

### Intervention uptake and exposure

Of patients who were offered to receive ARVs in their homes or at another community meeting point of their choosing (i.e., who were both clinically stable on ART and enrolled at a healthcare facility randomized to ARV community delivery), 87.4% decided to enroll in the program rather than remain in standard facility-based ART delivery. Over the course of the study period, a total of 151 HBCs (50 in Temeke, 45 in Kinondoni, and 56 in Ilala) conducted 3,039 household visits to 516 patients in or close to their homes. These patients received a mean of 5.9 (and a median of 6.0) home or community visits for ARV delivery and counseling during the trial period. Over the course of the study, 12 patients contacted the study team to inform them that the HBC had not delivered their ARV supply on time; these 12 patients were under the responsibility of a total of 4 HBCs.

### Virological failure

At the end of the study period (defined by the time of measurement of the study exit VL), 10.9% (95/872) and 9.7% (91/943) of patients were failing virologically in the control and intervention arm, respectively. Among those who had a suppressed VL at baseline, 4.3% (27/626) and 4.6% (31/671) were failing virologically at study exit in the control and intervention arm, respectively. Among those who received ARVs in or close to their homes, 5.7% (26/453) were failing virologically at study exit. When restricting the sample to those who had received ARVs in or close to their homes for at least 90 days prior to the date of VL measurement at study exit, 7.0% (24/345) were failing virologically.

The RR for virological failure comparing the patients in the intervention arm to the patients in the control arm was 0.89 (95% CI 0.63–1.25) in the primary (the unadjusted) model (Table 2). The upper bound of the 1-sided 95% CI for this RR was 1.18, and therefore below the non-inferiority margin of 1.45. When the sample was restricted to those patients with a suppressed VL (<1,000 copies/ml) at baseline—57.6% (440/764) of whom received ARV community delivery (as opposed 48.0% when including all ART patients at intervention facilities)—the RR was above 1, and the upper bound of the 1-sided 95% CI above the non-inferiority margin, in all models (S4 Table). In supplementary files, we also show results (i) when adjusting for follow-up time and time between the baseline and study exit VL measurement (S5 Table), (ii) when restricting the sample to those for whom the study exit VL was taken at least 200 days after enrollment into the trial (S6 Table), (iii) when restricting the sample to those for whom the study exit VL was taken at least 200 days after the baseline VL (or the baseline CD4 cell count) (S7 Table), and (iv) when adjusting for time on ART at baseline (S8 Table). The results were similar when using a VL threshold of ≥200 copies/ml to define virological failure (S9 Table).

**Table 2:** Effect of the intervention on the risk of virological failure^1^.

Statistic	Unadjusted^2^	Adjusted for baseline VL/CD4^3^	Adjusted for baseline VL/CD4, age, and sex^4^
*N*	1,815	1,551	1,494
RR (2-sided 95% CI)	0.89 (0.63–1.25)	0.96 (0.71–1.29)	1.00 (0.74–1.35)
*P* value^5^	0.489	0.766	0.998
One-sided 95% CI	0.00–1.18	0.00–1.23	0.00–1.28

^1^In all models, standard errors were adjusted for clustering at the healthcare facility level.

^2^This log-binomial model regressed virological failure (binary) on intervention arm (binary).

^3^This log-binomial model regressed virological failure (binary) on intervention arm (binary) and a binary indicator for whether the patient was in virological failure (VL <1,000 copies/ml or, if no VL value was available, CD4 cell count <350 cells/μl) at baseline.

^4^This log-binomial model regressed virological failure (binary) on intervention arm (binary), a binary indicator for whether the patient was in virological failure (VL <1,000 copies/ml or, if no VL value was available, CD4 cell count <350 cells/μl), age (continuous), and sex (binary).

^5^The *P* value tests the null hypothesis that the RR equals 1.0 with a significance level of alpha ≤0.05.

RR, risk ratio; VL, viral load.

The complier average causal effect (i.e., the effect of the ARV community delivery program on those who received ARVs in their homes) was not significantly different from 0 in all models (Table 3). The regression coefficients in Table 3 can be interpreted as the absolute difference in the probability (between 0 and 1) of virological failure in the intervention arm compared to the control arm. In the unadjusted model, receiving ARVs at home led to a 2.6 percentage point lower probability of being in virological failure at the end of the study period compared to being in the control arm. As supplementary files, we also show the complier average causal effect under different model specifications and sample restrictions (S10 Table), and when restricting the sample to those who had a suppressed VL at baseline (S11 Table).

**Table 3:** Estimates of the complier average causal effect using instrumental-variable regression^1^.

Statistic	Unadjusted	Adjusted for baseline VL/CD4^2^	Adjusted for baseline VL/CD4, age, and sex^3^
*N*	1,815	1,551	1,494
Coefficient (95% CI)	−0.026 (−0.099 to 0.047)	−0.006 (−0.063 to 0.052)	0.002 (−0.055 to 0.058)
*P* value^4^	0.487	0.848	0.951

^1^All models are 2-stage least squares regressions with the randomly assigned intervention status of the healthcare facilities participating in this trial as the instrumental variable for patients' potentially endogenous receipt of ARVs in their homes. Standard errors were adjusted for clustering at the healthcare facility level.

^2^This model included a binary indicator for whether the patient was in virological failure (VL <1,000 copies/ml or, if no VL value was available, CD4 cell count <350 cells/μl) at baseline as independent variable.

^3^This model included a binary indicator for whether the patient was in virological failure (VL <1,000 copies/ml or, if no VL value was available, CD4 cell count <350 cells/μl) at baseline, age (continuous), and sex (binary) as independent variables.

^4^The *P* value tests the null hypothesis that the coefficient equals 0.0 with a significance level of alpha ≤0.05.

VL, viral load.

### Patient healthcare expenditures

#### Cost to the patient of an ART visit

For the ART visit on the day of the study exit questionnaire, patients reported having incurred a median cost of TZS 800 (PPP$ 1.16) with an IQR of TZS 0 to 2,000 (PPP$ 0.00 to 2.89). The mean cost was TZS 3,445 (PPP$ 4.98), with a SD of TZS 16,795 (PPP$ 24.29). These costs included money lost from income-generating activities due to the time taken to attend care. The costs for the ART visit on the day of the baseline questionnaire were similar, with a median of TZS 800 (IQR TZS 0 to 3,000) and mean of TZS 5,831 (SD TZS 24,863), equal to PPP$ 1.16 (IQR PPP$ 0.00 to 4.34) and PPP$ 8.43 (SD PPP$ 35.96), respectively. The median and mean costs for an ART visit did not differ significantly when restricting the sample to those who were clinically stable on ART or those who received ARVs and counseling in or close to their homes. Among those who received ARVs and counseling in or close to their homes, 55% had previously been scheduled to pick up their ARVs from the facility once per month, and the remaining 45% once every 2 months. After enrollment in the trial, these patients were required to make only 1 ART clinic visit per year, resulting in an average of 8.3 fewer clinic visits per year. Using our figure for the cost of an ART visit in Dar es Salaam, simple extrapolation suggests that receiving ARV community delivery with a yearly checkup at the healthcare facility would reduce a patient’s cost to receive ART by a median of TZS 6,640 (PPP$ 9.61) per year.

#### Healthcare expenditures during the past 6 months

In the study exit questionnaire, only 36.6% of patients at intervention facilities and 6.5% at control facilities reported having attended a public-sector primary care clinic or private-sector doctor during the preceding 6 months, suggesting that most patients may have misunderstood the healthcare expenditure questions as excluding ART visits. S12 Table shows that there was no statistically significant difference in the mean or median healthcare expenditures of patients between the control and intervention arm.

### Percentage of ART patients shifted to community-based care

The percentage of all ART patients (regardless of whether they were eligible for enrollment into the trial or for receiving ARVs at home) at each intervention facility who received ARVs at home at least once varied from 0.3% to 19.0%, with a facility mean of 4.4% (Table 4).

**Table 4:** Percentage of ART patients at each intervention facility who received ARVs at home.

Facility name	Number of ART patients^1^	Number receiving ARVs at home	Percent of ART patients “shifted” into the community
Arafa Ugweno	202	6	3.0
Buza	215	18	8.4
Goba	177	17	9.6
Hananasif	530	18	3.4
Keko	79	15	19.0
Kigogo	347	10	2.9
Kimbiji	119	15	12.6
Kinyerezi	238	6	2.5
Kitunda	768	16	2.1
Mabibo	278	12	4.3
Mbagala Rangi Tatu	15,663	75	0.5
Mbezi	870	3	0.3
Mburahati	1,639	76	4.6
Mji mwema	161	11	6.8
Mongolandege	152	5	3.3
Mwenge	1,597	47	2.9
Pugu Kajiungeni	561	16	2.9
Tabata	2,193	33	1.5
Tabata NBC	249	6	2.4
Tambukareli	1,554	24	1.5
Tandale	2,951	20	0.7
Toa Ngoma	239	12	5.0
Vingunguti	1,865	42	2.3
Yombo Makangarawe	544	20	3.7

^1^Most of these ART patients were not enrolled in the trial because they did not reside in a neighborhood in the healthcare facility’s catchment area. The percentages shown in the last column of this table should, therefore, not be confused with uptake of the intervention among those who were eligible for the intervention.

ART, antiretroviral therapy; ARV, antiretroviral drug.

### Patient satisfaction with the intervention

**Fig 2:**
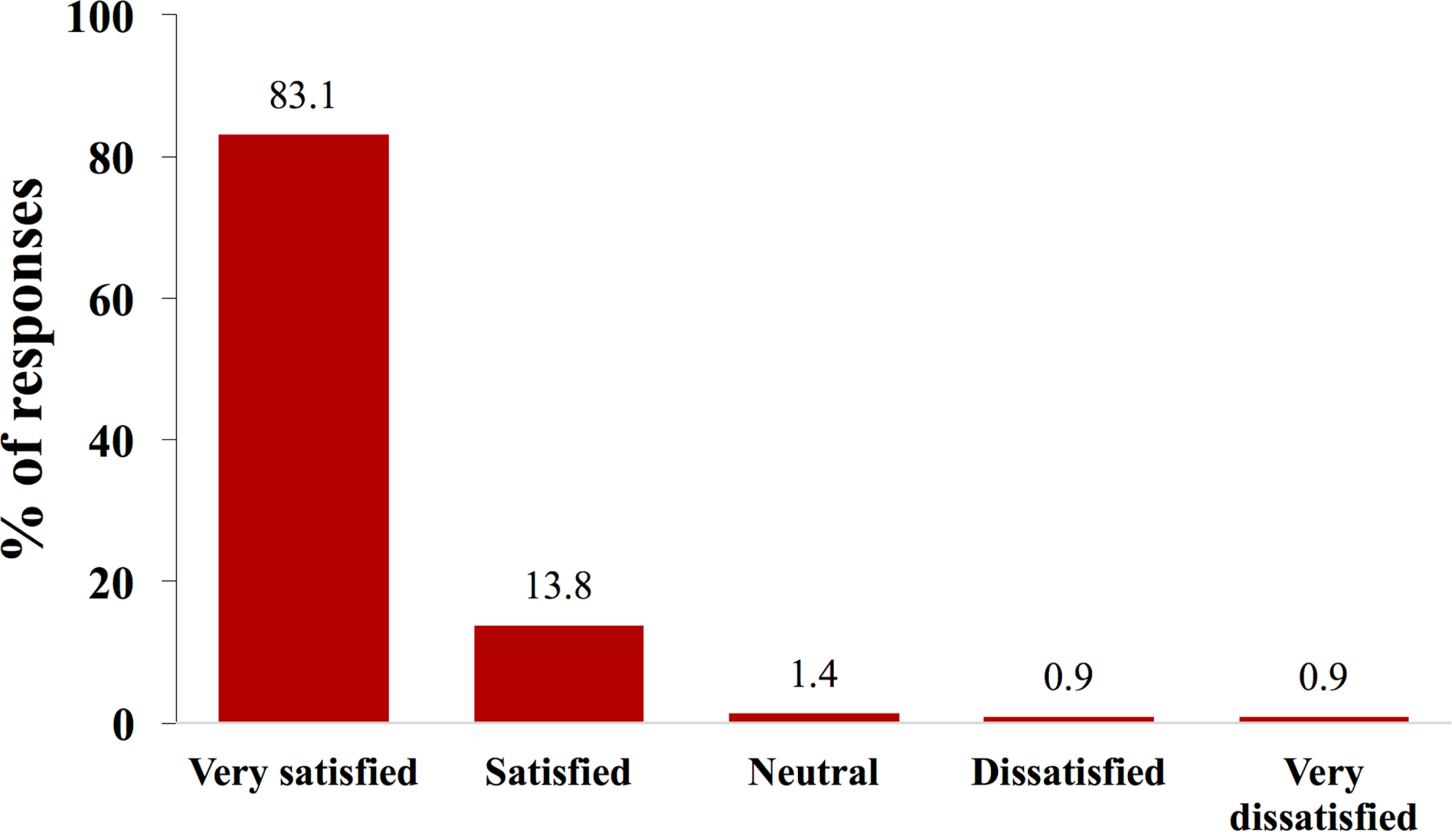
Distribution of responses to the question “Overall, how satisfied or dissatisfied are you with this program of home-based carers delivering HIV medicines into the community?”

**Fig 3:**
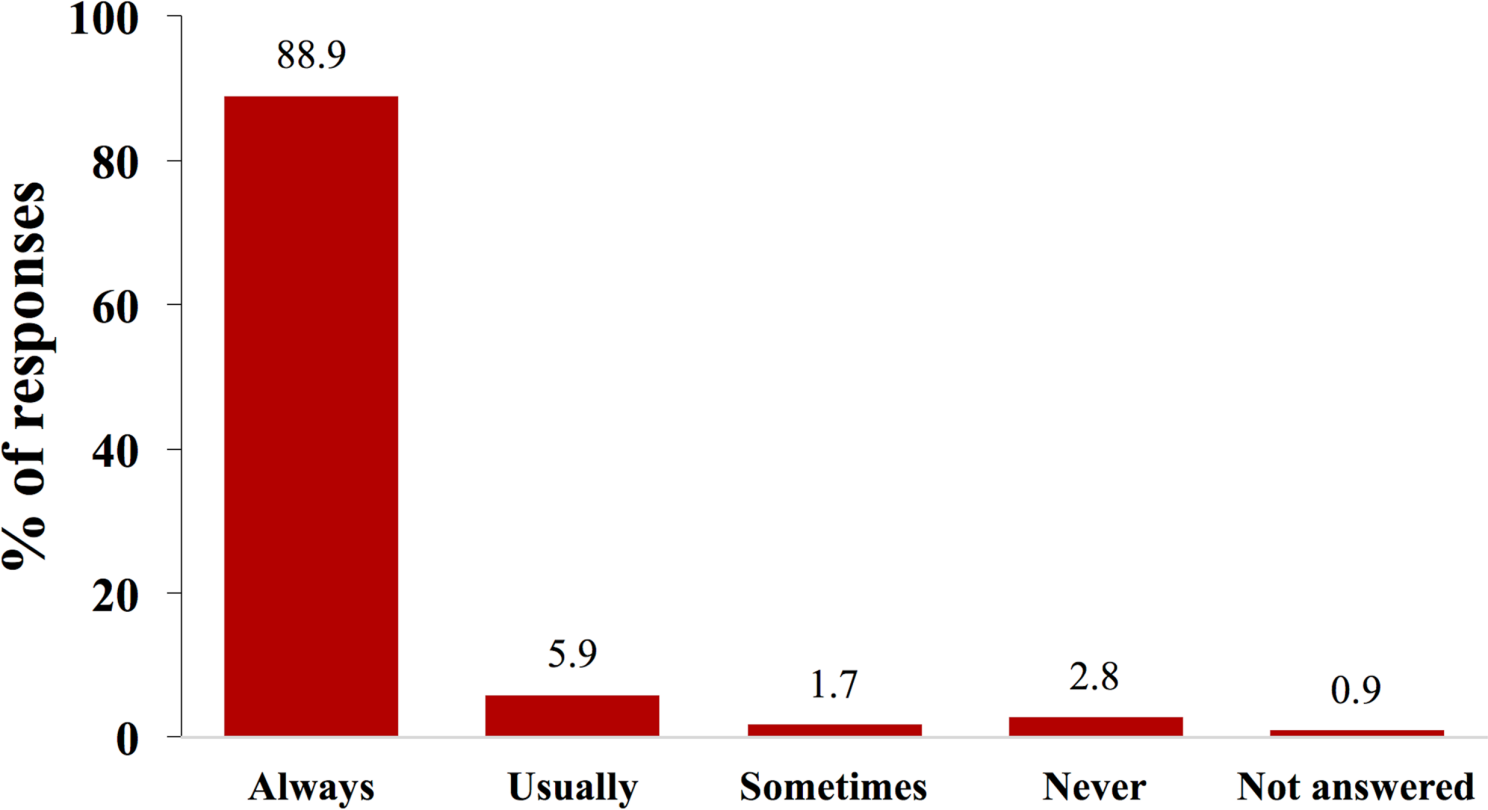
Distribution of responses to the question “Did the home-based carer deliver the HIV medicines on time?”

In the study exit questionnaire, 83.1% (295/355—the denominator is all those who received ARVs and counseling in or close to their homes and for whom data from the study exit questionnaire were available) reported being “very satisfied” with the program of receiving ARVs at home (Fig 2). In all, 88.7% (315/355) reported that the HBC always delivered the ARVs on time (Fig 3), and 2.0% (7/355) reported that they missed a dose of ARVs because the HBC did not deliver ARVs on time. In all, 96.3% (342/355) of those who received ARVs in or close to their homes reported that they would like to continue with this delivery model (rather than returning to standard facility-based care), and 99.7% (354/355) said they would recommend it to other communities. In total, 0.9% (3/355) of patients who received ARVs in or close to their homes reported that the program led to an unintentional disclosure of their HIV status to a third person.

### Challenges with data collection

We experienced 3 major challenges during data collection. First, most participants did not have a VL or CD4 cell count taken in the preceding 12 months in their clinical records at the time of the baseline questionnaire. Thus, the study team had to send a blood sample for VL testing to the laboratory. Receiving the results from the laboratory on these VL measurements took between 4 and 12 weeks. As a result, for most participants, the study team was not able to assess eligibility for the intervention until 1 to 3 months after the baseline questionnaire administration. Second, 417 participants did not return to the facility for their study exit assessment (and a clinical checkup)—136 of these participants were at control facilities and 281 at intervention facilities. For some of these individuals (as well as many individuals who had missing VL results for other reasons), we were able to retrieve their latest VL from the central health system database housed at MDH because Tanzania started implementing a yearly VL for all ART patients during the study period. The central health system database records all VLs taken at any healthcare facility in Dar es Salaam. Loss to follow-up in this report thus refers to participants who did not return to the healthcare facility for the study exit assessment and for whom we were unable to retrieve their VL from the central health system database. Third, we experienced difficulties in linking participants across our different study databases. The databases used in this study were a study logbook, in which the data collection team kept a list of all participants in the trial (as well as age and sex of the participant), the baseline questionnaire data, the baseline laboratory data, and the study exit questionnaire data. Out of the 2,172 participants in this study, we had all questionnaire data for 1,348 participants; 193 had only logbook data, 94 only logbook and baseline questionnaire data, 139 only logbook and baseline laboratory data, 271 only logbook, baseline questionnaire, and baseline laboratory data, and 127 only logbook and study exit questionnaire data. The proportion with complete data was similar between the 2 study arms—64.7% in the control arm and 59.6% in the intervention arm. These participant linking issues were responsible for the relatively high level of missingness in socio-demographic variables other than age and sex, and the lower sample size for the analysis of healthcare expenditures as compared to VL measurements. In addition to a relatively high number of participants not returning to the healthcare facility for the study exit assessment, the main cause of unsuccessful linking was that the data collectors entered neither the health system patient identifying number nor the study identifying number correctly into the tablet.

## Discussion

### Summary of findings

In this randomized controlled trial in Dar es Salaam, we investigated the effect of an ARV community delivery model—lay health workers delivering ARVs directly to patients’ homes or other meeting points in the community if the patient was stable on ART, and patients receiving standard facility-based care if they were unstable on ART—on the probability of virological failure and patients’ healthcare expenditures. We found that the ARV community delivery model performed at least as well in averting virological failure as standard facility-based ART.

Patient satisfaction with the program was high, and receiving ARVs in the community through HBCs is likely to save patients substantial amounts of time. However, 2 other envisaged benefits of the program—decongestion of healthcare facilities and reductions in patient healthcare expenditures—were minimal. The ARV community delivery model shifted only a small proportion of all ART patients at a healthcare facility from facility- to community-based care, so that it is unlikely to have had a noticeable effect on clinicians’ workload and waiting times at healthcare facilities. Regarding patient ART expenditures, the median cost of attending 1 ART visit for patients was low, and thus the extrapolated savings to patients from receiving ARVs in the community were small.

### Key considerations for policy-makers

Table 5 summarizes the key advantages and disadvantages of the ARV community delivery model tested in this trial. Regarding retention in care, ARV community delivery may improve long-term retention in care as only about 1 in 100 patients who received ARVs in or close to their homes were lost to care for reasons other than returning to standard facility-based care. However, because patients would continue to receive ARVs in or close to their homes regardless of whether they attended their once-a-year clinical checkup at the healthcare facility, they may miss these annual checkups, potentially reducing the clinical effectiveness of ART in the longer term. Implementation research needs to accompany any future scale-up of ARV community delivery to determine how the model performs over many years and how ART patients can be best motivated to attend their annual facility-based checkups. Moreover, in a routine scale-up of the ARV community delivery model, quality of care and clinical outcomes may be worse than those observed in this trial, because the intermittent presence of data collectors in the healthcare facilities that participated in this trial may have led to higher fidelity of implementation of the model and heightened attention to patient outcomes. The overall effect of ARV community delivery on per-patient costs is unclear at this point. Shifting ART from physicians and nurses to lay health workers will decrease the per-patient costs—if all other factors remain the same—because lay health workers earn less than physicians or nurses. However, some factors change when a health system moves from the facility- to the community-based ARV delivery model. Lay health workers may need more time to treat and care for ART patients because of the travel to the patient’s home and because each patient–health worker interaction may take longer in the home than in the facility-based setting—for instance, because patients need to find a private place before the interaction can take place, or because some of the 'rituals' that save time in the healthcare facility, such as minimal social conversation, may not be practiced in the home setting.

**Table 5:** Key considerations for local policy-makers regarding antiretroviral drug community delivery compared to standard facility-based care.

Positive	Neutral	Negative
• High patient satisfaction with the program • Slight reduction in patients’ ART expenses • Small reduction in ART patient volume at healthcare facilities • Likely time-saving for patients • Possibly higher long-term retention in ART	• Appears to result in equal (or better) clinical performance	• Risk of some patients not attending their yearly clinical checkup • May require the support of an additional staff member for successful implementation

ART, antiretroviral therapy.

### Increasing the proportion of patients who receive ARVs at home

An important limitation of the ARV community delivery program, as implemented in this study, is that it allowed for only a small proportion of ART patients at the study’s healthcare facilities to receive ARVs at home. Table 6 outlines possible ways of increasing this proportion. Because many ART patients in Dar es Salaam do not attend the healthcare facility closest to where they live, the main reason for the low enrollment in receiving ARVs at home was the eligibility criterion that a patient must reside in the facility’s catchment area. While removing this eligibility restriction would likely greatly increase the proportion of eligible ART patients, an important drawback is that delivering ARVs to patients’ homes would become logistically more complex and possibly more costly to implement. The HBCs would either have to travel across the entire city to deliver ARVs or a mechanism would have to be established by which an HBC affiliated with a different healthcare facility than the one the patient is attending (but which is closer to where the patient resides) would be tasked with delivering ARVs to the patient’s home. The former would be costly to implement due to the much higher transport costs compared to the current model. The latter would be unlikely to add substantial costs to the model tested in this study, but it would require fast and reliable communication across healthcare facilities. At the time of study conception, the study team felt that establishing such a mechanism would be too complex logistically to be successful. However, before the trial, the team was not aware of the extent to which restricting eligibility to patients living in the healthcare facility’s catchment area would impact on the proportion of ART patients that could be enrolled in the trial. Retrospectively, we, therefore, believe that it would have been worthwhile to more intensively investigate and pilot ways of delivering ARVs to the homes of patients living outside of their healthcare facility’s catchment area. Future implementation research should identify, design and test approaches for ARV community delivery outside of facility catchment areas.

**Table 6:** Possibilities to increase the proportion of ART patients who receive ARVs at home.

Possible modification	Advantages	Disadvantages
1. Removing the eligibility criterion that a patient must reside in the facility’s catchment area	Likely to lead to a large increase in enrollment	Logistically complex and possibly costly to implement
2. Increase the number of ART facilities in Dar es Salaam that have an affiliated team of HBCs	Would increase the number of healthcare facilities that can offer ARV community delivery	Cost of training and employing additional HBCs
3. Offer enrollment to all ART patients as long as they are able to meet up with the HBC within the facility’s catchment area	Likely to lead to a large increase in enrollment	Would not reduce transport time and costs for patients (only time lost from waiting at the healthcare facility)
4. Removing or relaxing^1^ the eligibility criterion that patients must be clinically stable on ART at enrollment	Would only lead to a small increase in enrollment	May be deemed to be unsafe
5. Do not use HBCs to deliver ARVs into the community (and instead use treatment supporters, delivery personnel on motorcycles, or other available cadres)	Depending on the details of the model, may lead to a large increase in enrollment	May be costly, would not build on a possible rapport between HBCs and their clients, and the non-HBC cadre may be unable to recognize symptoms/signs that require referral to a healthcare facility
6. Include pregnant women	Likely to lead to a substantial increase in enrollment	May reduce the frequency of clinical checkups that HIV-positive pregnant women receive

^1^For instance, instead of using the criterion that the most recent CD4 cell count must have been <350 cells/μl, one could use a threshold of <250 cells/μl.

ART, antiretroviral therapy; ARV, antiretroviral drug; HBC, home-based carer (the local cadre of lay health workers).

A second possibility to increase the number of patients enrolled in the ARV community delivery model is to expand the number of healthcare facilities in Dar es Salaam that have an affiliated team of lay health workers. At the time of study start, only about a third of the healthcare facilities that offered ART in Dar es Salaam also had a team of the local lay health worker cadre—the HBCs. A third possibility is to offer enrollment in the ARV community delivery program to all ART patients as long as they are able to meet up with the HBC within the facility’s catchment area. This would allow those who do not reside in the facility’s catchment area to still benefit from the program by foregoing the time spent waiting at the healthcare facility to pick up a new supply of ARVs. A fourth possibility, removing or relaxing the eligibility criterion that patients must be clinically stable on ART to receive ARVs in their homes, would only lead to a small increase in enrollment. In this study, removing the clinical stability criterion for eligibility would have led to an increase in enrollment of only 15.7%. As a fifth possibility, one could imagine a model that delivers ARVs to patients’ homes (or other meeting points in the community) but instead of HBCs uses another lay cadre for this purpose, such as HIV treatment supporters. This approach would, however, have the disadvantage that it would not harness the rapport that many HBCs have developed with members of the communities they serve. In addition, the non-HBC cadre may have less training or experience than the HBCs to recognize symptoms and signs that require referral to a nurse or physician. Lastly, enrolling pregnant women living with HIV would likely substantially increase enrollment numbers, but such a model would need to be carefully designed to avoid a reduction in antenatal care attendance.

### Implementation lessons for future trials

This trial suffered from a number of implementation challenges. While we are certainly not the first to have faced such difficulties, and there are excellent resources on implementing randomized trials in resource-poor settings (e.g., Glennerster and Takavarasha [23]), it is our view that other researchers planning pragmatic health services trials in resource-poor settings might benefit from our experience. Specifically, we have drawn the following implementation lessons from this trial for our future work. First, relying on data collectors to correctly enter long unique identifying codes for patients into study registers and tablets should be avoided wherever possible, such as by trying to automate the process (for instance, with the use of bar codes). Second, collecting data in the minimum number of datasets needed to accomplish the task at hand will minimize linkage problems across datasets. Third, in future work, we will endeavor to devise measures early to reduce the possibility of bias from loss to follow-up. One approach in this regard could be to randomize the level of encouragement (e.g., the number of phone calls or level of monetary compensation) that patients receive to return to the healthcare facility for the study exit assessment, which would create an instrument for loss to follow-up that could be exploited to correct for attrition based on both observable and unobservable characteristics [24]. Lastly, in the case of non-inferiority trials, an extensive piloting period might be useful to verify to what degree the assumptions about the envisaged benefits of the intervention are likely to hold true, and thus whether a non-inferiority design is justified.

### Limitations

This study has several limitations. First, only a relatively small proportion of study participants at intervention facilities received ARVs in the community through the HBCs (48.0% [453/943] of participants at intervention facilities were enrolled in the program of receiving ARVs through the HBCs, and 41.3% [345/835] received ARVs through the HBCs for at least 90 days before the study exit VL measurement was taken). The primary analysis as per our study protocol, however, included all ART patients at a healthcare facility who resided in the facility’s catchment area because patients at intervention facilities who remain in facility-based care may indirectly benefit from some patients receiving ARVs at home through the envisaged decongestion of the healthcare facility. All else remaining equal, the lower the proportion of study participants at intervention facilities who receive ARVs at home, the more similar the ARV community delivery model is to the standard of care and thus the more likely the intervention is to appear to have no effect. Therefore, if patients receiving ARVs at home through HBCs had a worse virological outcome than if they had remained in standard facility-based care, this study may have found the ARV community delivery model to be non-inferior based on this “dilution” of the effect of receiving ARVs at home. To try to ascertain whether our non-inferiority conclusion is partly due to this dilution effect, we show the results when restricting the sample in secondary analyses to only those patients who had a suppressed VL at baseline—58.9% (395/671) of whom received ARVs at home (S4 Table). The RR in the unadjusted model among this sample was 1.07, but the upper bound of the 1-sided 95% CI (1.75) was above the non-inferiority margin (1.45), largely because this study was not powered to determine non-inferiority for this smaller sample of patients. Overall, it is important to note that dilution could only explain some part of our results, because dilution can never lead to an improvement in outcomes in the intervention arm, which is what we observe. We also calculated the complier average causal effect (i.e., the effect of the ARV community delivery model on only those who received ARVs at home) and found that the point estimates were generally close to 0 (although the CIs were fairly wide) (Table 3).

Second, the proportion of patients LTFU was not only relatively high, but also substantially larger in the intervention than in the control arm. This raises the concern that even if those LTFU in the intervention arm had the same probability of failing virologically at study exit as those LTFU in the control arm, this trial might have falsely concluded that ARV community delivery performs at least as well as the standard of care simply if those LTFU (regardless of study arm) had a higher probability of virological failure than those included in the analysis. This was the case at baseline (S3 Table) and, to the degree that baseline virological failure predicts virological failure at study exit, was thus likely also the case at study exit. However, even if we assumed the most extreme scenario, namely that 100% of those LTFU (regardless of study arm) were in virological failure at study exit, the unadjusted RR (i.e., our primary analysis) would have been 1.16, with the upper bound of a 1-sided 95% CI being 1.32, which is still below the margin of non-inferiority of 1.45 (S1 Text). Our conclusions are, therefore, robust to this source of bias. A more likely scenario in which bias from attrition could have changed our conclusions is that those LTFU in the intervention arm may have had a higher probability of failing virologically at study exit than those LTFU in the control arm. However, the fact that those who subsequently were LTFU in the intervention arm were less—not more—likely to be failing virologically at baseline than those LTFU in the control arm (S3 Table) suggests that (under the assumption that baseline virological failure predicts virological failure at study exit) this scenario is unlikely. Nonetheless, while our results appear to be robust to bias from attrition, it is possible that we might have reached a different conclusion if this study had had no attrition.

Third, it can be argued that the level at which the margin of non-inferiority was set (here, a RR of 1.45) is arbitrary. We have, however, adhered to best practices in setting this non-inferiority margin by involving relevant policy-makers in the decision, considering the margin set in similar studies conducted prior to this trial, and specifying the non-inferiority margin in our study registration. Nonetheless, the somewhat arbitrary nature of setting a non-inferiority margin is a limitation of non-inferiority trials in general [25], and thus it also applies to this trial.

Fourth, patients in the control arm appear to not have included ART visits when answering questions on health service utilization in the preceding 6 months. A more extensive piloting phase might have uncovered this misperception among respondents prior to the trial, which would have allowed us to word the relevant questions differently. In addition, qualitative work accompanying this trial could have shed further light on whether, and if so why, respondents did not include ART visits in these answers. However, given the low costs incurred from attending ART, it is unlikely that our estimates of patient healthcare expenditures during the preceding 6 months would have been substantially different had patients included ART visits in their response. In our view, a more important limitation of this study with regards to patient healthcare expenditures was that only those living close to the healthcare facility—and thus likely facing the lowest transport costs to get to the healthcare facility—were eligible for enrollment. It is therefore possible that the cost savings to patients would have been higher had all ART patients at a healthcare facility been eligible to enroll, regardless of the neighborhood in which they lived. Similarly, the cost savings would likely have been higher in more rural settings, where the average distance from a patient's home to the nearest healthcare facility is typically far longer than in urban settings.

Fifth, this study excluded pregnant women, and thus the trial results cannot be generalized to PMTCT care. Lastly, with patients receiving ARVs at home for an average of 226 days, we were unable to assess the longer-term safety of the ARV community delivery model.

## Conclusion

As implemented in this trial, with roughly 40% of patients in the intervention arm receiving ARVs at home and 60% remaining in standard facility-based care, ARV community delivery performed at least as well as the standard of care regarding the critical health indicator of virological failure. ARV community delivery did not significantly reduce patient healthcare expenditures, but satisfaction with the program of receiving ARVs at home was high. In addition, receiving ARVs at home is likely to save patients substantial amounts of time and may reduce government health expenditures per ART patient. It is our view that with modifications to allow a larger proportion of ART patients at healthcare facilities to enroll, the ARV community delivery model can serve as an important alternative for ART delivery. The model holds particular promise for settings where—relative to demand—human and physical resources for ART are increasingly scarce. As the model is scaled up to serve increasingly large populations in the future, accompanying implementation research can ensure that issues arising due to the greater scale and longer time horizons than those in our trial are quickly detected and addressed.

## Supporting information

S1 Checklist CONSORT checklist. (DOC)

S1 Table Characteristics of the clusters. (DOCX)

S2 Table Activities over the study period. (DOCX)

S3 Table Sample characteristics comparing those lost to follow-up with those included in the analysis. (DOCX)

S4 Table Risk of virological failure among those who were clinically stable at baseline. (DOCX)

S5 Table Risk of virological failure adjusting for follow-up time and time between baseline and study exit viral load measurement. (DOCX)

S6 Table Risk of virological failure among those for whom the study exit viral load was taken at least 200 days after enrollment into the trial. (DOCX)

S7 Table Risk of virological failure among those for whom the study exit viral load was taken at least 200 days after the baseline viral load (or the baseline CD4 cell count). (DOCX)

S8 Table Effect of the intervention on the risk of virological failure when adjusting for time on ART at baseline. (DOCX)

S9 Table Effect of the intervention on the risk of virological failure when defining virological failure as a viral load ≥200 copies/ml. (DOCX)

S10 Table Robustness checks of the complier average causal effect. (DOCX)

S11 Table Complier average causal effect among those who had a suppressed viral load at baseline. (DOCX)

S12 Table Impact of the intervention on patient healthcare expenditures during the 6 months preceding study exit. (DOCX)

S1 Text Sensitivity of the results to loss to follow-up. (DOCX)

## Abbreviations

ART: antiretroviral therapy
ARV: antiretroviral drug
HBC: home-based carer
IQR: interquartile range
ITT: intent-to-treat
LTFU: lost to follow-up
MDH: Management and Development for Health
PMTCT: prevention of mother-to-child transmission of HIV
PPP$: purchasing-power-parity-adjusted dollars
RR: risk ratio
SD: standard deviation
SSA: sub-Saharan Africa
TZS: Tanzanian Shillings
VL: viral load
WHO: World Health Organization

## References

[1] GBD 2016 DALYs and HALE Collaborators. Global, regional, and national disability-adjusted life-years (DALYs) for 333 diseases and injuries and healthy life expectancy (HALE) for 195 countries and territories, 1990–2016: a systematic analysis for the Global Burden of Disease Study 2016. Lancet. 2017;390(10100):1260–344. 10.1016/S0140-6736(17)32130-X 28919118 PMC5605707

[2] Bärnighausen T, Welz T, Hosegood V, Bätzing-Feigenbaum J, Tanser F, Herbst K, et al Hiding in the shadows of the HIV epidemic: obesity and hypertension in a rural population with very high HIV prevalence in South Africa. J Hum Hypertens. 2008;22:236–9. 10.1038/sj.jhh.1002308 18046436

[3] Hontelez JA, de Vlas SJ, Baltussen R, Newell ML, Bakker R, Tanser F, et al The impact of antiretroviral treatment on the age composition of the HIV epidemic in sub-Saharan Africa. AIDS. 2012;26(Suppl 1):S19–30. 22781175 10.1097/QAD.0b013e3283558526 PMC3886374

[4] Joint United Nations Programme on HIV/AIDS. Ending AIDS: progress towards the 90–90–90 targets Geneva: Joint United Nations Programme on HIV/AIDS; 2017.

[5] World Health Organization. Consolidated guidelines on the use of antiretroviral drugs for treating and preventing HIV infection—recommendations for a public health approach Geneva: World Health Organization; 2016. 27466667

[6] Hontelez JA, Chang AY, Ogbuoji O, de Vlas SJ, Bärnighausen T, Atun R. Changing HIV treatment eligibility under health system constraints in sub-Saharan Africa: investment needs, population health gains, and cost-effectiveness. AIDS. 2016;30(15):2341–50. 10.1097/QAD.0000000000001190 27367487 PMC5017264

[7] World Health Organization. Consolidated guidelines on the use of antiretroviral drugs for treating and preventing HIV infection Geneva: World Health Organization; 2013. 24716260

[8] Chimbindi N, Bor J, Newell ML, Tanser F, Baltusen R, Hontelez J, et al Time and money: the true costs of health care utilization for patients receiving ‘free’ HIV/TB care and treatment in rural KwaZulu-Natal. J Acquir Immune Defic Syndr. 2015;70(2):e52–60. 10.1097/QAI.0000000000000728 26371611 PMC4748708

[9] Ware NC, Wyatt MA, Geng EH, Kaaya SF, Agbaji OO, Muyindike WR, et al Toward an understanding of disengagement from HIV treatment and care in sub-Saharan Africa: a qualitative study. PLoS Med. 2013;10(1):e1001369; 10.1371/journal.pmed.1001369 23341753 PMC3541407

[10] Jaffar S, Amuron B, Foster S, Birungi J, Levin J, Namara G, et al Rates of virological failure in patients treated in a home-based versus a facility-based HIV-care model in Jinja, southeast Uganda: a cluster-randomised equivalence trial. Lancet. 2009;374(9707):2080–9. 10.1016/S0140-6736(09)61674-3 19939445 PMC2806484

[11] United Nations. The world’s cities in 2016 New York: United Nations; 2016.

[12] Geldsetzer P, Francis JM, Ulenga N, Sando D, Lema IA, Mboggo E, et al The impact of community health worker-led home delivery of antiretroviral therapy on virological suppression: a non-inferiority cluster-randomized health systems trial in Dar es Salaam, Tanzania. BMC Health Serv Res. 2017;17(1):160 10.1186/s12913-017-2032-7 28228134 PMC5322683

[13] Tanzania Commission for AIDS, Zanzibar AIDS Commission, National Bureau of Statistics, Office of the Chief Government Statistician, ICF International. Tanzania HIV/AIDS and Malaria Indicator Survey 2011–2012: key findings. Dar es Salaam: Tanzania Commission for AIDS; 2013.

[14] Tanzanian National AIDS Control Programme. National guidelines for the management of HIV and AIDS 5th edition Dar es Salaam: Ministry of Health and Social Welfare; 2015.

[15] Bärnighausen T, Bloom DE, Humair S. Human resources for treating HIV/AIDS: needs, capacities, and gaps. AIDS Patient Care STDs. 2007;21(11):799–812. 10.1089/apc.2007.0193 17944556

[16] Jones PM. SSI: Stata module to estimate sample size for randomized controlled trials Boston: Boston College Department of Economics; 2010 [cited 2015 Nov 24]. Available from: https://ideas.repec.org/c/boc/bocode/s457150.html.

[17] Hemming K, Marsh J. A menu-driven facility for sample-size calculations in cluster randomized controlled trials. Stata J. 2013;13(1):114–35.

[18] Barnhart D, Hertzmark E, Liu E, Mungure E, Muya AN, Sando D, et al Intra-cluster correlation estimates for HIV-related outcomes from care and treatment clinics in Dar es Salaam, Tanzania. Contemp Clin Trials Commun. 2016;4:161–9. 10.1016/j.conctc.2016.09.001 27766318 PMC5066589

[19] A’Court C, Stevens R, Heneghan C. Against all odds? Improving the understanding of risk reporting. Br J Gen Pract. 2012;62(596):e220–3. 10.3399/bjgp12X630223 22429441 PMC3289830

[20] Duflo E, Glennerster R, Kremer M. Using randomization in development economics research: a toolkit In: Schultz T, Strauss J, editors. Handbook of development economics. 4th edition North Holland: Elsevier Science; 2007.

[21] Heß S. Randomization inference with Stata: a guide and software. Stata J. 2017;17(3):630–51.

[22] Athey S, Imbens G. The econometrics of randomized experiments. arXiv; 2016 [cited 2018 Aug 30]. Available from: https://arxiv.org/abs/1607.00698.

[23] Glennerster R, Takavarasha K. Running randomized evaluations: a practical guide Princeton: Princeton University Press; 2013.

[24] Huber M. Identification of average treatment effects in social experiments under alternative forms of attrition. J Educ Behav Stat. 2012;37(3):443–74.

[25] Piaggio G, Elbourne DR, Pocock SJ, Evans SJ, Altman DG. Reporting of noninferiority and equivalence randomized trials: extension of the CONSORT 2010 statement. JAMA. 2012;308(24):2594–604. 10.1001/jama.2012.87802 23268518

