# Supplementary material for: Community delivery of antiretroviral drugs: A non-inferiority cluster-randomized pragmatic trial in Dar es Salaam, Tanzania
Source: PLoS Med. 2018 Sep 19;15(9):e1002659. doi: 10.1371/journal.pmed.1002659 (PMC6145501; doi:10.1371/journal.pmed.1002659)
Supplement: S1 Table — (DOCX) [file pmed.1002659.s002.docx]

# **S1 Table. Characteristics of the clusters**

| **Name of healthcare facility** | **Type of healthcare facility** | **Municipality** | **No. of patients currently on ART** | **No. of HBCs in cluster** |
| --- | --- | --- | --- | --- |
| ***ARV community delivery*** |  |  |  |  |
| 1. Mbagala Rangi Tatu | Hospital | Temeke | 15,663 | 3 |
| 2. Tambukareli | Dispensary | Temeke | 1,554 | 12 |
| 3. Yombo Makangarawe | Dispensary | Temeke | 544 | 2 |
| 4. Toa Ngoma | Dispensary | Temeke | 239 | 12 |
| 5. Buza | Dispensary | Temeke | 215 | 5 |
| 6. Arafa Ugweno | Dispensary | Temeke | 202 | 3 |
| 7. Mji mwema | Dispensary | Temeke | 161 | 8 |
| 8. Kimbiji | Dispensary | Temeke | 119 | 6 |
| 9. Keko | Dispensary | Temeke | 79 | 4 |
| 10. Tandale | Dispensary | Kinondoni | 2,951 | 9 |
| 11. Mburahati | Dispensary | Kinondoni | 1,639 | 11 |
| 12. Mwenge | Dispensary | Kinondoni | 1,597 | 5 |
| 13. Mbezi | Dispensary | Kinondoni | 870 | 11 |
| 14. Hananasif | Dispensary | Kinondoni | 530 | 7 |
| 15. Kigogo | Dispensary | Kinondoni | 347 | 3 |
| 16. Mabibo | Dispensary | Kinondoni | 278 | 9 |
| 17. Goba | Dispensary | Kinondoni | 177 | 4 |
| 18. Tabata | Health Centre | Ilala | 2,193 | 10 |
| 19. Vingunguti | Health Centre | Ilala | 1,865 | 7 |
| 20. Kitunda | Health Centre | Ilala | 768 | 10 |
| 21. Pugu Kajiungeni | Dispensary | Ilala | 561 | 11 |
| 22. Tabata NBC | Dispensary | Ilala | 249 | 10 |
| 23. Kinyerezi | Dispensary | Ilala | 238 | 13 |
| 24. Mongolandege | Dispensary | Ilala | 152 | 16 |
|  | ***Total:*** |  | ***33,191^1^*** | ***191*** |
| ***Standard of care*** |  |  |  |  |
| 1. Temeke | Hospital | Temeke | 17,409 | 3 |
| 2. Kigamboni | Health Centre | Temeke | 2,879 | 9 |
| 3. Mbagala Round Table | Dispensary | Temeke | 850 | 5 |
| 4. Maji Matitu | Dispensary | Temeke | 540 | 7 |
| 5. Kichemchem | Dispensary | Temeke | 211 | 3 |
| 6. Kingugi | Dispensary | Temeke | 166 | 6 |
| 7. Sandali | Dispensary | Temeke | 148 | 9 |
| 8. Kibada | Dispensary | Temeke | 109 | 8 |
| 9. Kisarawe II | Dispensary | Temeke | 63 | 5 |
| 10. Magomeni | Health Center | Kinondoni | 2,361 | 8 |
| 11. Kimara | Dispensary | Kinondoni | 2,270 | 5 |
| 12. Bunju | Dispensary | Kinondoni | 1,595 | 7 |
| 13. Kawe | Dispensary | Kinondoni | 844 | 5 |
| 14. Kijitonyama | Dispensary | Kinondoni | 750 | 8 |
| 15. Kinondoni | Hospital | Kinondoni | 396 | 5 |
| 16. Makuburi | Dispensary | Kinondoni | 256 | 4 |
| 17. Ununio | Dispensary | Kinondoni | 115 | 6 |
| 18. Mnazi Mmoja Health Centre | Health Centre | Ilala | 3,650 | 4 |
| 19. Chanika | Health Centre | Ilala | 1,413 | 12 |
| 20. Segerea | Health Centre | Ilala | 678 | 8 |
| 21. Kiwalani | Dispensary | Ilala | 519 | 13 |
| 22. Gerezani | Dispensary | Ilala | 350 | 2 |
| 23. Majohe | Dispensary | Ilala | 220 | 10 |
| 24. Mvuti | Dispensary | Ilala | 185 | 16 |
|  | ***Total:*** |  | ***37,977^1^*** | ***168*** |

Abbreviations: ART = antiretroviral therapy; HBC = home-based carer; No. = number;

^1^ This is *not* the expected number of participants as many ART patients did not reside in the cluster (i.e., in the area surrounding the healthcare facility), and were therefore not eligible for this trial.

Acknowledgement: This table has been adapted from [1].

**References:**

1. Geldsetzer P, Francis JM, Ulenga N, Sando D, Lema IA, Mboggo E, et al. The impact of community health worker-led home delivery of antiretroviral therapy on virological suppression: a non-inferiority cluster-randomized health systems trial in Dar es Salaam, Tanzania. BMC Health Serv Res. 2017;17(1):160. Epub 2017/02/24. doi: 10.1186/s12913-017-2032-7. PubMed PMID: 28228134.
