# Supplementary material for: Community delivery of antiretroviral drugs: A non-inferiority cluster-randomized pragmatic trial in Dar es Salaam, Tanzania
Source: PLoS Med. 2018 Sep 19;15(9):e1002659. doi: 10.1371/journal.pmed.1002659 (PMC6145501; doi:10.1371/journal.pmed.1002659)
Supplement: S2 Table — (DOCX) [file pmed.1002659.s003.docx]

# **S2 Table. Activities over the study period**

|  | **STUDY PERIOD** | | | | | | | | | | | | | | | | | | | |
| --- | --- | --- | --- | --- | --- | --- | --- | --- | --- | --- | --- | --- | --- | --- | --- | --- | --- | --- | --- | --- |
| **Month** | **1** | **2** | **3** | **4** | **5** | **6** | **7** | **8** | **9** | **10** | **11** | **12** | **13** | **14** | **15** | **16** | **17** | **18** | **19** | **20** |
| **ENROLMENT**  ***Temeke municipality:*** | | | | | | | | | | | | |  |  |  |  |  |  |  |  |
| Eligibility screen | X | X | X | X | X |  |  |  |  |  |  |  |  |  |  |  |  |  |  |  |
| Informed consent | X | X | X | X | X |  |  |  |  |  |  |  |  |  |  |  |  |  |  |  |
| ***Kinondoni municipality:*** | | | | | | | | | | | | | | | | | | | | |
| Eligibility screen |  |  |  |  |  | X | X | X |  |  |  |  |  |  |  |  |  |  |  |  |
| Informed consent |  |  |  |  |  | X | X | X |  |  |  |  |  |  |  |  |  |  |  |  |
| ***Ilala municipality:*** | | | | | | | | | | | | | | | | | | | | |
| Eligibility screen |  |  |  |  |  |  |  |  | X | X | X |  |  |  |  |  |  |  |  |  |
| Informed consent |  |  |  |  |  |  |  |  | X | X | X |  |  |  |  |  |  |  |  |  |
| **BASELINE ASSESSMENTS**  ***Temeke municipality*** | | | | | | | | | | | | | | | | | | | | |
| Baseline questionnaire | X | X | X | X | X |  |  |  |  |  |  |  |  |  |  |  |  |  |  |  |
| VL^1^ | X | X | X | X | X |  |  |  |  |  |  |  |  |  |  |  |  |  |  |  |
| ***Kinondoni municipality*** | | | | | | | | | | | | | | | | | | | | |
| Baseline questionnaire |  |  |  |  |  | X | X | X |  |  |  |  |  |  |  |  |  |  |  |  |
| VL^1^ |  |  |  |  |  | X | X | X |  |  |  |  |  |  |  |  |  |  |  |  |
| ***Ilala*** ***municipality*** | | | | | | | | | | | | | | | | | | | | |
| Baseline questionnaire |  |  |  |  |  |  |  |  | X | X | X |  |  |  |  |  |  |  |  |  |
| VL^1^ |  |  |  |  |  |  |  |  | X | X | X |  |  |  |  |  |  |  |  |  |
|  |  |  |  |  |  |  |  |  |  |  |  |  |  |  |  |  |  | | | |
| **INTERVENTIONS**  ***Temeke municipality*** | | | | | | | | | | | | |  |  |  |  |  | | | |
| ARV community delivery |  | X | X | X | X | X | X | X | X | X | X | X | X |  |  |  |  |  |  |  |
| ***Kinondoni municipality*** | | | | | | | | | | | | | | | | | | | | |
| ARV community delivery |  |  |  |  |  |  | X | X | X | X | X | X | X | X | X |  |  |  |  |  |
|  |  |  |  |  |  |  |  |  |  |  |  |  |  |  |  |  |  | | | |
| ***Ilala municipality*** | | | | | | | | | | | | | | | | | | | | |
| ARV community delivery |  |  |  |  |  |  |  |  |  | X | X | X | X | X | X | X | X |  |  |  |
|  |  |  |  |  |  |  |  |  |  |  |  |  |  |  |  |  |  | | | |
| **EXIT ASSESSMENTS**  ***Temeke municipality*** | | | | | | | | | | | | |  |  |  |  |  |  |  |  |
| Study exit questionnaire |  |  |  |  |  |  |  |  |  |  |  |  | X | X | X | X | X | X | X | X |
| VL |  |  |  |  |  |  |  |  |  |  |  |  | X | X | X | X | X | X | X | X |
| ***Kinondoni municipality*** | | | | | | | | | | | | | | | | | | | | |
| Study exit questionnaire |  |  |  |  |  |  |  |  |  |  |  |  |  |  | X | X | X | X | X | X |
| VL |  |  |  |  |  |  |  |  |  |  |  |  |  |  | X | X | X | X | X | X |
| ***Ilala municipality*** | | | | | | | | | | | | | | | | | | | | |
| Study exit questionnaire |  |  |  |  |  |  |  |  |  |  |  |  |  |  |  |  | X | X | X | X |
| VL |  |  |  |  |  |  |  |  |  |  |  |  |  |  |  |  | X | X | X | X |

**Abbreviations:** BMI = Body Mass Index

^1^ VL was measured at enrolment if the participant has not had a VL measurement in the preceding 12 months. Otherwise, the most current VL measurement will be recorded.

Acknowledgement: This table has been adapted from [1].

1. GBD 2016 DALYs and HALE Collaborators. Global, regional, and national disability-adjusted life-years (DALYs) for 333 diseases and injuries and healthy life expectancy (HALE) for 195 countries and territories, 1990-2016: a systematic analysis for the Global Burden of Disease Study 2016. Lancet. 2017;390(10100):1260-344. PMID: 28919118
