# Supplementary material for: Community delivery of antiretroviral drugs: A non-inferiority cluster-randomized pragmatic trial in Dar es Salaam, Tanzania
Source: PLoS Med. 2018 Sep 19;15(9):e1002659. doi: 10.1371/journal.pmed.1002659 (PMC6145501; doi:10.1371/journal.pmed.1002659)
Supplement: S3 Table — (DOCX) [file pmed.1002659.s004.docx]

# **S3 Table. Sample characteristics comparing those LTFU with those included in the analysis**

|  | **Control** | | **Intervention** | |
| --- | --- | --- | --- | --- |
|  | *Not LTFU* | *LTFU* | *Not LTFU* | *LTFU* |
| n | 872 | 137 | 943 | 220 |
| Male, n (%) | 129 (15.4) | 22 (19.3) | 203 (22.2) | 21 (15.4) |
| Age in years, mean (SD) | 38.7 (8.6) | 38.9 (9.4) | 40.5 (9.4) | 39.1 (9.9) |
| Age group, n (%) |  |  |  |  |
| 18-25 years | 41 (4.9) | 6 (5.4) | 32 (3.5) | 10 (7.6) |
| 26-35 years | 260 (31.2) | 34 (30.6) | 259 (28.5) | 39 (29.8) |
| 36-45 years | 371 (44.6) | 48 (43.2) | 384 (42.3) | 52 (39.7) |
| 46-55 years | 129 (15.5) | 16 (14.4) | 171 (18.8) | 22 (16.8) |
| 56-65 years | 25 (3.0) | 6 (5.4) | 53 (5.8) | 7 (5.3) |
| >65 years | 6 (0.7) | 1 (0.9) | 9 (1.0) | 1 (0.8) |
| Education, n (%) |  |  |  |  |
| <Primary school | 26 (4.3) | 3 (4.2) | 66 (9.8) | 6 (6.2) |
| Primary school | 473 (77.7) | 51 (71.8) | 512 (76.1) | 76 (78.4) |
| Secondary school | 110 (18.1) | 17 (23.9) | 95 (14.1) | 15 (15.5) |
| Married, n (%) | 237 (35.8) | 20 (27.4) | 334 (44.3) | 39 (39.4) |
| Time on ART in days, mean (SD) | 1059 (952) | 1199 (1097) | 1407 (1171) | 1438 (1183) |
| Time on ART, n (%) |  |  |  |  |
| <90 days | 57 (9.6) | 8 (11.3) | 48 (7.5) | 7 (7.7) |
| 90-179 days | 34 (5.7) | 6 (8.5) | 19 (3.0) | 4 (4.4) |
| 180-364 days | 73 (12.3) | 3 (4.2) | 58 (9.1) | 4 (4.4) |
| 1 to <3 years | 210 (35.3) | 25 (35.2) | 202 (31.6) | 33 (36.3) |
| 3 to <5 years | 109 (18.3) | 12 (16.9) | 121 (18.9) | 14 (15.4) |
| ≥ 5 years | 112 (18.8) | 17 (23.9) | 191 (29.9) | 29 (31.9) |
| Disclosed HIV status to at least one person, n (%) | 542 (88.4) | 67 (93.1) | 625 (92.0) | 90 (92.8) |
| VL ≥ 1,000 copies/ml or CD4 <350 cells/μl, n (%) | 132 (17.4) | 29 (28.2) | 122 (15.4) | 23 (19.8) |
| Received ARVs at home, n (%) | 0 (0.0) | 0 (0.0) | 453 (48.0) | 63 (28.6) |

Abbreviations: ART=antiretroviral therapy; ARV=antiretroviral drug; SD=standard deviation; ml=milliliter; μl=microliter.
