# Supplementary material for: Community delivery of antiretroviral drugs: A non-inferiority cluster-randomized pragmatic trial in Dar es Salaam, Tanzania
Source: PLoS Med. 2018 Sep 19;15(9):e1002659. doi: 10.1371/journal.pmed.1002659 (PMC6145501; doi:10.1371/journal.pmed.1002659)
Supplement: S4 Table — (DOCX) [file pmed.1002659.s005.docx]

# **S4 Table. Risk of virological failure among those who were clinically stable at baseline**

Note: Clinical stability for the purpose of the table below was defined as having a suppressed (>1,000 copies/ml) VL at baseline or, if no VL was available at baseline, a CD4-cell count >350 cells/microliter.

|  | **N** | **RR (95% CI)^1^** | **P^2^** | **One-sided 95% CI** |
| --- | --- | --- | --- | --- |
| *Model 1*^3^ | 1,297 | 1.07 (0.60 - 1.92) | 0.818 | 0.00 - 1.75 |
| *Model 2*^4^ | 1,240 | 1.17 (0.63 - 2.18) | 0.624 | 0.00 - 1.97 |
| *Model 3*^5^ | 1,051 | 1.21 (0.64 - 2.28) | 0.555 | 0.00 - 2.06 |
| *Model 4*^6^ | 1,051 | 1.22 (0.64 - 2.34) | 0.544 | 0.00 - 2.11 |
| *Model 5*^7^ | 1,011 | 1.30 (0.67 - 2.51) | 0.442 | 0.00 - 2.25 |

Abbreviations: RR=relative risk; CI=CI

^1^ In all models, standard errors were adjusted for clustering at the healthcare facility level.

^2^ The p-value tests the null hypothesis that the RR equals 1.0 with a significance level of alpha ≤0.05.

^3^ This log-binomial model regressed virological failure (binary) onto intervention arm (binary).

^4^ This log-binomial model regressed virological failure (binary) onto intervention arm (binary), and the time in days between the enrolment into the trial and the study exit VL measurement (continuous).

^5^ This log-binomial model regressed virological failure (binary) onto intervention arm (binary), and the time in days between the baseline VL (or CD4-cell count) and the study exit VL measurement (continuous).

^6^ This log-binomial model regressed virological failure (binary) onto intervention arm (binary), the time in days between the enrolment into the trial and the study exit VL measurement (continuous), and the time in days between the baseline VL (or CD4-cell count) and the study exit VL measurement (continuous).

^7^ This log-binomial model regressed virological failure (binary) onto intervention arm (binary), the time in days between the enrolment into the trial and the study exit VL measurement (continuous), the time in days between the baseline VL (or CD4-cell count) and the study exit VL measurement (continuous), age (continuous), and sex (binary).
