# Supplementary material for: Community delivery of antiretroviral drugs: A non-inferiority cluster-randomized pragmatic trial in Dar es Salaam, Tanzania
Source: PLoS Med. 2018 Sep 19;15(9):e1002659. doi: 10.1371/journal.pmed.1002659 (PMC6145501; doi:10.1371/journal.pmed.1002659)
Supplement: S5 Table — (DOCX) [file pmed.1002659.s006.docx]

# **S5 Table. Risk of virological failure adjusting for follow-up time, and time between baseline and endline viral load**

|  | **N** | **RR (95% CI)^1^** | **P^2^** | **One-sided 95% CI** |
| --- | --- | --- | --- | --- |
| *Model 1*^3^ | 1,475 | 1.01 (0.73 – 1.39) | 0.964 | 0.00 - 1.32 |
| *Model 2*^4^ | 1,266 | 1.05 (0.75 – 1.47) | 0.791 | 0.00 - 1.39 |
| *Model 3*^5^ | 1,266 | 1.02 (0.72 - 1.44) | 0.912 | 0.00 - 1.37 |

Abbreviations: RR=relative risk; CI=CI

^1^ In all models, standard errors were adjusted for clustering at the healthcare facility level.

^2^ The p-value tests the null hypothesis that the RR equals 1.0 with a significance level of alpha ≤0.05.

^3^ This log-binomial model regressed virological failure (binary) onto intervention arm (binary), a binary indicator for whether the participant was in virological failure (or, if no VL was available, had a CD4-cell count <350 cells/microliter) at baseline, and the time in days between the enrolment into the trial and the study exit VL measurement (continuous).

^4^ This log-binomial model regressed virological failure (binary) onto intervention arm (binary), a binary indicator for whether the participant was in virological failure (or, if no VL was available, had a CD4-cell count <350 cells/microliter) at baseline, and the time in days between the baseline VL (or CD4-cell count) and the study exit VL measurement (continuous).

^5^ This log-binomial model regressed virological failure (binary) onto intervention arm (binary), a binary indicator for whether the participant was in virological failure (or, if no VL was available, had a CD4-cell count <350 cells/microliter) at baseline, the time in days between the enrolment into the trial and the study exit VL measurement (continuous), and the time in days between the baseline VL (or CD4-cell count) and the study exit VL measurement (continuous).
