# Supplementary material for: Community delivery of antiretroviral drugs: A non-inferiority cluster-randomized pragmatic trial in Dar es Salaam, Tanzania
Source: PLoS Med. 2018 Sep 19;15(9):e1002659. doi: 10.1371/journal.pmed.1002659 (PMC6145501; doi:10.1371/journal.pmed.1002659)
Supplement: S6 Table — (DOCX) [file pmed.1002659.s007.docx]

# **S6 Table. Risk of virological failure among those for whom the study exit viral load was taken at least 200 days after enrolment into the trial**

|  | **N** | **RR (95% CI)^1^** | **P^2^** | **One-sided 95% CI** |
| --- | --- | --- | --- | --- |
| *Model 1*^3^ | 1,567 | 0.89 (0.61 - 1.28) | 0.526 | 0.00 - 1.21 |
| *Model 2*^4^ | 1,344 | 0.95 (0.69 - 1.31) | 0.766 | 0.00 - 1.24 |
| *Model 3*^5^ | 1,268 | 1.00 (0.69 - 1.43) | 0.983 | 0.00 - 1.35 |
| *Model 4*^6^ | 1,097 | 1.04 (0.74 - 1.48) | 0.810 | 0.00 - 1.40 |
| *Model 5*^7^ | 1,097 | 1.01 (0.70 - 1.46) | 0.959 | 0.00 - 1.38 |
| *Model 6*^8^ | 1,048 | 1.14 (0.83 - 1.57) | 0.418 | 0.00 - 1.49 |

Abbreviations: RR=relative risk; CI=CI

^1^ In all models, standard errors were adjusted for clustering at the healthcare facility level.

^2^ The p-value tests the null hypothesis that the RR equals 1.0 with a significance level of alpha ≤0.05.

^3^ This log-binomial model regressed virological failure (binary) onto intervention arm (binary).

^4^ This log-binomial model regressed virological failure (binary) onto intervention arm (binary) and a binary indicator for whether the participant was in virological failure (or, if no VL was available, had a CD4-cell count <350 cells/microliter) at baseline.

^5^ This log-binomial model regressed virological failure (binary) onto intervention arm (binary), a binary indicator for whether the participant was in virological failure (or, if no VL was available, had a CD4-cell count <350 cells/microliter) at baseline, and the time in days between the enrolment into the trial and the study exit VL measurement (continuous).

^6^ This log-binomial model regressed virological failure (binary) onto intervention arm (binary), a binary indicator for whether the participant was in virological failure (or, if no VL was available, had a CD4-cell count <350 cells/microliter) at baseline, and the time in days between the baseline VL (or CD4-cell count) and the study exit VL measurement (continuous).

^7^ This log-binomial model regressed virological failure (binary) onto intervention arm (binary), a binary indicator for whether the participant was in virological failure (or, if no VL was available, had a CD4-cell count <350 cells/microliter) at baseline, the time in days between the enrolment into the trial and the study exit VL measurement (continuous), and the time in days between the baseline VL (or CD4-cell count) and the study exit VL measurement (continuous).

^8^ This log-binomial model regressed virological failure (binary) onto intervention arm (binary), a binary indicator for whether the participant was in virological failure (or, if no VL was available, had a CD4-cell count <350 cells/microliter) at baseline, the time in days between the enrolment into the trial and the study exit VL measurement (continuous), the time in days between the baseline VL (or CD4-cell count) and the study exit VL measurement (continuous), age (continuous), and sex (binary).
