# Supplementary material for: Community delivery of antiretroviral drugs: A non-inferiority cluster-randomized pragmatic trial in Dar es Salaam, Tanzania
Source: PLoS Med. 2018 Sep 19;15(9):e1002659. doi: 10.1371/journal.pmed.1002659 (PMC6145501; doi:10.1371/journal.pmed.1002659)
Supplement: S7 Table — (DOCX) [file pmed.1002659.s008.docx]

# **S7 Table. Risk of virological failure among those for whom the study exit viral load was taken at least 200 days after the baseline viral load (or CD4-cell count)**

|  | **N** | **RR (95% CI)^1^** | **P^2^** | **One-sided 95% CI** |
| --- | --- | --- | --- | --- |
| *Model 1*^3^ | 1,711 | 0.86 (0.60 - 1.23) | 0.413 | 0.00 - 1.16 |
| *Model 2*^4^ | 1,447 | 0.93 (0.68 - 1.26) | 0.633 | 0.00 - 1.20 |
| *Model 3*^5^ | 1,371 | 0.98 (0.69 - 1.37) | 0.890 | 0.00 - 1.30 |
| *Model 4*^6^ | 1,162 | 1.02 (0.72 - 1.45) | 0.915 | 0.00 - 1.37 |
| *Model 5*^7^ | 1,162 | 0.99 (0.68 - 1.43) | 0.950 | 0.00 - 1.35 |
| *Model 6*^8^ | 1,115 | 1.11 (0.78 - 1.57) | 0.565 | 0.00 - 1.48 |

Abbreviations: RR=relative risk; CI=CI

^1^ In all models, standard errors were adjusted for clustering at the healthcare facility level.

^2^ The p-value tests the null hypothesis that the RR equals 1.0 with a significance level of alpha ≤0.05.

^3^ This log-binomial model regressed virological failure (binary) onto intervention arm (binary).

^4^ This log-binomial model regressed virological failure (binary) onto intervention arm (binary) and a binary indicator for whether the participant was in virological failure (or, if no VL was available, had a CD4-cell count <350 cells/microliter) at baseline.

^5^ This log-binomial model regressed virological failure (binary) onto intervention arm (binary), a binary indicator for whether the participant was in virological failure (or, if no VL was available, had a CD4-cell count <350 cells/microliter) at baseline, and the time in days between the enrolment into the trial and the study exit VL measurement (continuous).

^6^ This log-binomial model regressed virological failure (binary) onto intervention arm (binary), a binary indicator for whether the participant was in virological failure (or, if no VL was available, had a CD4-cell count <350 cells/microliter) at baseline, and the time in days between the baseline VL (or CD4-cell count) and the study exit VL measurement (continuous).

^7^ This log-binomial model regressed virological failure (binary) onto intervention arm (binary), a binary indicator for whether the participant was in virological failure (or, if no VL was available, had a CD4-cell count <350 cells/microliter) at baseline, the time in days between the enrolment into the trial and the study exit VL measurement (continuous), and the time in days between the baseline VL (or CD4-cell count) and the study exit VL measurement (continuous).

^8^ This log-binomial model regressed virological failure (binary) onto intervention arm (binary), a binary indicator for whether the participant was in virological failure (or, if no VL was available, had a CD4-cell count <350 cells/microliter) at baseline, the time in days between the enrolment into the trial and the study exit VL measurement (continuous), the time in days between the baseline VL (or CD4-cell count) and the study exit VL measurement (continuous), age (continuous), and sex (binary).
