# Supplementary material for: Community delivery of antiretroviral drugs: A non-inferiority cluster-randomized pragmatic trial in Dar es Salaam, Tanzania
Source: PLoS Med. 2018 Sep 19;15(9):e1002659. doi: 10.1371/journal.pmed.1002659 (PMC6145501; doi:10.1371/journal.pmed.1002659)
Supplement: S8 Table — (DOCX) [file pmed.1002659.s009.docx]

**S8 Table. Effect of the intervention on the risk of virological failure when adjusting for time on ART at baseline.^1^**

|  | **Adjusted for baseline VL/CD4^2^** | **Adjusted for baseline VL/CD4, age, and sex^3^** |
| --- | --- | --- |
| *N* | 1,234 | 1,233 |
| *RR (two-sided 95% CI)* | 0.96 (0.72 – 1.28) | 0.98 (0.73 – 1.30) |
| *P^4^* | 0.774 | 0.873 |
| *One-sided 95% CI* | 0.00 – 1.22 | 0.00 – 1.24 |

Abbreviations: VL=viral load; RR=risk ratio; CI=confidence interval.

^1^ In all models, standard errors were adjusted for clustering at the healthcare facility level.

^2^ This log-binomial model regressed virological failure (binary) onto intervention arm (binary) and a categorical variable for the time that the participant had been on ART at baseline (<90 days, 90 to 179 days, 180 to 364 days, 1 to <3 years, 3 to <5 years, ≥5 years).

^3^ This log-binomial model regressed virological failure (binary) onto intervention arm (binary), a categorical variable for the time that the participant had been on ART at baseline (<90 days, 90 to 179 days, 180 to 364 days, 1 to <3 years, 3 to <5 years, ≥5 years), age (continuous), and sex (binary).

^4^ The p-value tests the null hypothesis that the RR equals 1.0 with a significance level of alpha ≤0.05.
