# Supplementary material for: Community delivery of antiretroviral drugs: A non-inferiority cluster-randomized pragmatic trial in Dar es Salaam, Tanzania
Source: PLoS Med. 2018 Sep 19;15(9):e1002659. doi: 10.1371/journal.pmed.1002659 (PMC6145501; doi:10.1371/journal.pmed.1002659)
Supplement: S9 Table — (DOCX) [file pmed.1002659.s010.docx]

**S9 Table. Effect of the intervention on the risk of virological failure when defining virological failure as a viral load ≥200 copies/millilitre.^1^**

|  | **Unadjusted^2^** | **Adjusted for baseline VL/CD4^3^** | **Adjusted for baseline VL/CD4, age, and sex^4^** |
| --- | --- | --- | --- |
| *N* | 1,815 | 1,551 | 1,494 |
| *RR (two-sided 95% CI)* | 0.96 (0.76 – 1.21) | 1.00 (0.82 – 1.22) | 1.05 (0.86 – 1.27) |
| *P^5^* | 0.721 | 0.988 | 0.672 |
| *One-sided 95% CI* | 0.00 – 1.16 | 0.00 – 1.18 | 0.00 – 1.23 |

Abbreviations: VL=viral load; RR=risk ratio; CI=confidence interval.

^1^ In all models, standard errors were adjusted for clustering at the healthcare facility level.

^2^ This log-binomial model regressed virological failure (binary) onto intervention arm (binary).

^3^ This log-binomial model regressed virological failure (binary) onto intervention arm (binary) and a binary indicator for whether the participant was in virological failure (or, if no VL was available, had a CD4-cell count <350 cells/microliter) at baseline.

^4^ This log-binomial model regressed virological failure (binary) onto intervention arm (binary), a binary indicator for whether the participant was in virological failure (or, if no VL was available, had a CD4-cell count <350 cells/microliter), age (continuous), and sex (binary).

^5^ The p-value tests the null hypothesis that the RR equals 1.0 with a significance level of alpha ≤0.05.
