# Supplementary material for: Community delivery of antiretroviral drugs: A non-inferiority cluster-randomized pragmatic trial in Dar es Salaam, Tanzania
Source: PLoS Med. 2018 Sep 19;15(9):e1002659. doi: 10.1371/journal.pmed.1002659 (PMC6145501; doi:10.1371/journal.pmed.1002659)
Supplement: S10 Table — (DOCX) [file pmed.1002659.s011.docx]

**S10 Table. Robustness checks of the complier average causal effect**^1^

|  | **Unadjusted** | **Adjusted for baseline VL/CD4^2^** | **Adjusted for baseline VL/CD4, age, and sex^3^** |
| --- | --- | --- | --- |
| *Must have ≥90 days between enrolment into receiving ARVs at home and the study exit VL measurement to be considered to have received ARVs at home* | | | |
| *N* | 1815 | 1551 | 1494 |
| *Coefficient (95% CI)* | -0.037 (-0.142 - 0.067) | -0.008 (-0.090 - 0.074) | 0.003 (-0.078 - 0.083) |
| *P^4^* | 0.484 | 0.847 | 0.951 |
| *Must have ≥180 days between enrolment into receiving ARVs at home and the study exit VL measurement to be considered to have received ARVs at home* | | | |
| *N* | 1815 | 1551 | 1494 |
| *Coefficient (95% CI)* | -0.052 (-0.197 - 0.093) | -0.011 (-0.125 - 0.102) | 0.004 (-0.108 - 0.115) |
| *P^4^* | 0.482 | 0.847 | 0.951 |
| *Only includes those for whom the study exit VL was taken at least 200 days after the baseline VL (or CD4-cell count) AND must have ≥90 days between enrolment into receiving ARVs at home and the study exit VL measurement to be considered to have received ARVs at home* | | | |
| *N* | 1711 | 1447 | 1397 |
| *Coefficient (95% CI)* | -0.043 (-0.145 - 0.058) | -0.014 (-0.092 - 0.065) | -0.003 (-0.080 - 0.074) |
| *P^4^* | 0.403 | 0.736 | 0.934 |
| *Only includes those for whom the study exit VL was taken at least 200 days after the baseline VL (or CD4-cell count) AND must have ≥180 days between enrolment into receiving ARVs at home and the study exit VL measurement to be considered to have received ARVs at home* | | | |
| *N* | 1711 | 1447 | 1397 |
| *Coefficient (95% CI)* | -0.060 (-0.199 - 0.079) | -0.019 (-0.127 - 0.089) | -0.004 (-0.110 - 0.101) |
| *P^4^* | 0.401 | 0.735 | 0.934 |

Abbreviations: VL=VL; CD4=cluster of differentiation 4 cell count; ARV = antiretroviral drugs; CI=CI

^1^ All models are two-stage least squares regression models with the endogenous independent variable being a binary indicator for whether the participant received ARVs at, or close to, their home and the instrument being a binary indicator for study arm. Standard errors were adjusted for clustering at the healthcare facility level.

^2^ This model included a binary indicator for whether the participant was in virological failure (or, if no VL was available, had a CD4-cell count <350 cells/microliter) at baseline as independent variable.

^3^ This model included a binary indicator for whether the participant was in virological failure (or, if no VL was available, had a CD4-cell count <350 cells/microliter) at baseline, age (continuous), and sex (binary) as independent variables.

^4^ The p-value tests the null hypothesis that the coefficient equals 0.0 with a significance level of alpha ≤0.05.
