# Supplementary material for: Community delivery of antiretroviral drugs: A non-inferiority cluster-randomized pragmatic trial in Dar es Salaam, Tanzania
Source: PLoS Med. 2018 Sep 19;15(9):e1002659. doi: 10.1371/journal.pmed.1002659 (PMC6145501; doi:10.1371/journal.pmed.1002659)
Supplement: S11 Table — (DOCX) [file pmed.1002659.s012.docx]

**S11 Table. Complier average causal effect among those who had a suppressed viral load at baseline**^1^

|  | **Unadjusted** | **Adjusted for age and sex^2^** |
| --- | --- | --- |
| *N* | 1297 | 1255 |
| *Coefficient (95% CI)* | 0.005 (-0.038 - 0.049) | 0.009 (-0.036 - 0.053) |
| *P^3^* | 0.815 | 0.708 |
| *Must have ≥90 days between enrolment into receiving ARVs at home and the study exit VL measurement to be considered to have received ARVs at home* | | |
| *N* | 1297 | 1255 |
| *Coefficient (95% CI)* | 0.007 (-0.055 - 0.070) | 0.012 (-0.051 - 0.076) |
| *P^3^* | 0.815 | 0.709 |
| *Must have ≥180 days between enrolment into receiving ARVs at home and the study exit VL measurement to be considered to have received ARVs at home* | | |
| *N* | 1297 | 1255 |
| *Coefficient (95% CI)* | 0.0104 (-0.077 - 0.098) | 0.017 (-0.072 - 0.106) |
| *P^3^* | 0.815 | 0.709 |
| *Only includes those for whom the study exit VL was taken at least 200 days after the baseline VL (or CD4-cell count) AND must have ≥90 days between enrolment into receiving ARVs at home and the study exit VL measurement to be considered to have received ARVs at home* | | |
| *N* | 1219 | 1182 |
| *Coefficient (95% CI)* | 0.008 (-0.056 - 0.072) | 0.014 (-0.051 - 0.079) |
| *P^3^* | 0.811 | 0.676 |
| *Only includes those for whom the study exit VL was taken at least 200 days after the baseline VL (or CD4-cell count) AND must have ≥180 days between enrolment into receiving ARVs at home and the study exit VL measurement to be considered to have received ARVs at home* | | |
| *N* | 1219 | 1182 |
| *Coefficient (95% CI)* | 0.011 (-0.078 - 0.010) | 0.019 (-0.071 - 0.109) |
| *P^3^* | 0.811 | 0.676 |

Abbreviations: CD4=cluster of differentiation 4 cell count; CI=CI

^1^ All models are two-stage least squares regression models with the endogenous independent variable being a binary indicator for whether the participant received ARVs at home and the instrument being a binary indicator for study arm. Standard errors were adjusted for clustering at the healthcare facility level.

^2^ This model included age (continuous) and sex (binary) as independent variables.

^3^ The p-value tests the null hypothesis that the coefficient equals 0.0 with a significance level of alpha ≤0.05.
