# Supplementary material for: Community delivery of antiretroviral drugs: A non-inferiority cluster-randomized pragmatic trial in Dar es Salaam, Tanzania
Source: PLoS Med. 2018 Sep 19;15(9):e1002659. doi: 10.1371/journal.pmed.1002659 (PMC6145501; doi:10.1371/journal.pmed.1002659)
Supplement: S12 Table — (DOCX) [file pmed.1002659.s013.docx]

# **S12 Table. Impact of the intervention on patient healthcare expenditures during the preceding six months**

| **Inference based on the mean (TZS)** | | | | **Inference based on the median (TZS)** | | | |
| --- | --- | --- | --- | --- | --- | --- | --- |
| *Control*  *(95% CI)* | *Intervention*  *(95% CI)* | *Coefficient*  *(95% CI)^1^* | *P^2^* | *Control*  *(IQR)* | *Intervention (IQR)* | *Coefficient*  *(95% CI)^3^* | *P^2^* |
| 2312 (1863 – 2762) | 4483 (2890 – 6077) | 1529 (138 - 2920) | 0.092 | 800 (800-2000) | 800 (0 – 3000) | -400 (-2368 - 1568) | 0.076 |

Abbreviations: TZS = Tanzanian Shillings; IQR = interquartile range

^1^ As obtained from an ordinary least squares regression of cost onto an indicator for intervention or control facility and indicator variables for each facility pair (as used in the matched-pair randomization). Standard errors were adjusted for clustering at the facility level.

^2^ As obtained from randomization inference with 10,000 repetitions.

^3^ As obtained from a median regression of cost onto an indicator for intervention or control facility and indicator variables for each facility pair (as used in the matched-pair randomization). Standard errors were adjusted for clustering at the facility level.
