# Supplementary material for: Community delivery of antiretroviral drugs: A non-inferiority cluster-randomized pragmatic trial in Dar es Salaam, Tanzania
Source: PLoS Med. 2018 Sep 19;15(9):e1002659. doi: 10.1371/journal.pmed.1002659 (PMC6145501; doi:10.1371/journal.pmed.1002659)
Supplement: S1 Text — (DOCX) [file pmed.1002659.s014.docx]

**S1 Text. Sensitivity of the results to loss to follow-up**

This supplementary file explains the (simple) calculation conducted for the following statement in the limitations section of the Discussion: “However, even if we assumed the most extreme scenario, namely that 100% of those LTFU (regardless of study arm) were in virological failure at study exit, the unadjusted RR (i.e., our primary analysis) would have been 1.16 with the upper bound of a one-sided 95% CI being 1.32, which is still below the margin of non-inferiority of 1.45.”

As detailed in the results, 137 and 220 participants were lost to follow-up (LTFU) in the control and intervention arm, respectively. For the statement above, we assumed all participants who were LTFU to have been in virological failure at the end of the study period. This assumption would increase the proportion of participants in virological failure at study exit in the control arm to 0.2299 ([95 + 137]/1,009) and in the intervention arm to 0.2674 ([91 + 220] /1163). The point estimate for the risk ratio (RR) of virological failure comparing intervention to control arm thus becomes 0.2674 / 0.2299 = 1.16. The upper bound of the corresponding one-sided 95% confidence interval was determined using a log-binomial model adjusting standard errors for clustering at the level of a healthcare facility.
